# Altered RBC deformability in diabetes: clinical characteristics and RBC pathophysiology

**DOI:** 10.1186/s12933-024-02453-2

**Published:** 2024-10-18

**Authors:** Ifechukwude Ebenuwa, Pierre-Christian Violet, Hongbin Tu, Casey Lee, Nicholas Munyan, Yu Wang, Mahtab Niyyati, Kartick Patra, Kenneth J. Wilkins, Nermi Parrow, Mark Levine

**Affiliations:** 1 Molecular and Clinical Nutrition Section, Digestive Diseases Branch, Intramural Research Program, National Institute of Diabetes and Digestive and Kidney Diseases, National Institutes of Health, Bethesda, MD 20892 USA; 2 Biostatistics Program, Office of Clinical Research Support, Office of the Director, National Institute of Diabetes and Digestive and Kidney Diseases, National Institutes of Health, Bethesda, MD 20892 USA

## Abstract

**Background:**

Reduced red blood cell deformability (RBCD) is associated with diabetic vascular complications, but early pathophysiological RBC changes and predictive demographic and clinical factors in populations with diabetes are unclear. An understanding of early diabetes-specific RBC changes associated with impaired RBCD is essential in investigating mechanisms that predispose to diabetic vascular complications.

**Methods:**

We conducted an outpatient cross-sectional study of participants in a well-controlled diabetes cohort (N81) and nondiabetic controls (N78) at the National Institutes of Health. First, between-group differences in RBCD measures were assessed with shear stress-gradient ektacytometry. Differences in structural RBC parameters were assessed using osmotic gradient ektacytometry and NaCl osmotic fragility. Functional RBC changes were assessed using hemoglobin-oxygen dissociation: p50.

**Results:**

All shear-stress gradient RBCD measures were significantly altered in the diabetes cohort vs. nondiabetic controls, even after adjustment for confounding covariates (*p* < 0.001). Adjusted for diabetes-status and demographic factors, significant predictors of reduced RBCD included older age, Black race, male gender, hyperglycemia, and vascular complications (all *p* < 0.05). Reduced RBCD was also associated with aberrant osmotic-gradient parameters, with a left-shift on osmotic gradient profile indicative of dehydrated RBCs in diabetes. A structure-function relationship was observed with reduced RBCD associated with reduced osmotic fragility (*P* < 0.001) and increased hemoglobin-oxygen dissociation (*P* < 0.01).

**Conclusions:**

Findings suggest impaired RBCD incurs similar demographic and clinical risk factors as diabetic vascular disease, with early pathophysiological RBC changes indicative of disordered RBC hydration in diabetes. Findings provide strong evidence for disordered oxygen release as a functional consequence of reduced RBCD.

*Clinical trial number*: NCT00071526.

**Supplementary Information:**

The online version contains supplementary material available at 10.1186/s12933-024-02453-2.

## Introduction

Diabetic vascular complications cause morbidity and mortality in millions of people worldwide and have been associated with reduced red blood cell deformability (RBCD): the ability of RBCs to change their shape in the microvasculature [1–8]. Reduced RBCD may impair microvascular flow and oxygen-delivering capacity–the hallmark of diabetic microvascular complications [6, 9–12]. With advances in ektacytometry, RBCD and other rheological parameters can be accurately and reproducibly captured by rheometry coupled to a gradient of applied incremental shear stress at fixed osmolality (shear stress gradient ektacytometry) or with varying osmolality at a fixed shear stress (osmotic gradient ektacytometry) [13–15].

Although shear stress gradient ektacytometry studies have revealed reduced RBCD in populations with diabetes [1, 2, 4], the clinical and RBC features associated with reduced RBCD in diabetes remain poorly characterized. Shear stress gradient ektacytometry includes several raw and derived measures [13, 14], but the RBCD parameters most applicable to populations with diabetes are unclear, as varied and inconsistent measures have been used [1, 2, 4]. Additionally, there are minimal characterizations of how demographic and diabetes-related clinical factors contribute to RBCD changes in diabetes. In contrast to hematological disorders with defined phenotypes, diabetes is a complex syndrome influenced by physiological changes such as aging, and pathological comorbidities such as hyperglycemia, insulin resistance and obesity. An understanding of how these diabetes-related clinical parameters contribute to RBCD changes is lacking, and necessary for understanding the clinical relevance and applicability of RBCD in diabetes.

The mechanisms associated with reduced RBCD in diabetes are also unclear. Some have hypothesized that reduced RBCD in diabetes would result in increased RBC rigidity, osmotic fragility, RBC hemolysis and impaired oxygen release in the peripheral tissues, which over time, would result in impaired perfusion and ultimately vascular complications [6, 16–18]. However, these hypotheses are primarily based on studies of hematological disorders and have yet to be investigated in cohorts with diabetes, using advanced ektacytometry techniques [19–22]. Osmotic gradient ektacytometry provides rheological information that could account for altered RBCD, such as RBC membrane characteristics, water content and osmotic fragility [15, 23]. Surprisingly, osmotic gradient ektacytometry has not been applied to diabetes. An understanding of early diabetes-specific RBC changes associated with impaired RBCD is essential in investigating mechanisms that predispose to diabetic vascular complications.

This study had three objectives. The first was to characterize differences in RBCD parameters in cohorts with and without diabetes, using shear stress-gradient ektacytometry, and accounting for associated demographic and clinical factors. The second was to characterize rheological RBC changes associated with impaired RBCD in diabetes, using osmotic gradient ektacytometry. The third was to investigate hypothesis linking impaired RBCD to changes in osmotic fragility and altered hemoglobin-oxygen release in participants with vs. without diabetes. We hypothesized that altered RBCD in diabetes would be associated with demographic and clinical factors, and diabetes-specific differences in osmotic fragility and altered hemoglobin-oxygen release. Hypotheses were tested in a cohort of participants with diabetes and nondiabetic controls, using a cross-sectional study design.

## Methods

### Clinical study design

The study was conducted at the National Institutes of Health Clinical Center (NIHCC) under protocol 04-DK-0021 (clinical trial number: NCT00071526) and approved by the Institutional Review Boards of NIDDK, NIH. Participants were recruited from the Washington DC area community from November 2013 to 2017, using recruitment flyers and advertisements displayed across NIHCC, community, social media, and online registries. Inclusion criteria were males and females aged 18–65, with type 1 or type 2 diabetes, and healthy nondiabetic controls. Exclusion criteria included presence of an acute or chronic illness (other than complications of diabetes mellitus and/or obesity-related syndrome), alcohol abuse, active tobacco use and pregnancy. Consecutive sampling approach was used, with separate screening and study visits at the National Institutes of Health. All participants were recruited and studied on a rolling basis, in accordance with NIH protocol 04-DK-0021. Data were obtained from clinical history, anthropometric measurements and fasting laboratory studies obtained on morning of outpatient study visit. Fasting blood and urine chemistry measurements were performed, using standard laboratory methods, by the Department of Laboratory Medicine Clinical Laboratory Improvement Amendments (CLIA) ID number 21D0665373, Clinical Research Center, NIH. Blood samples for all RBC physiological studies (described below) were collected into heparinized tubes (Fisher Scientific) and processed within 4 h of collection. A standardized sample collection process was used, consistent with the guidelines for hemorheological laboratory techniques [24]. 

### Shear stress gradient ektacytometry

RBCD was measured with the Laser-assisted Optical Rotational Red Cell Analyzer (LORRCA) (R&R Mechatronics, Hoorn, Netherlands). To quantify RBCD, the diffraction pattern of RBCs was measured over shear stress gradient ranging from 0.3 to 30 Pa [14, 15]. Ektacytometry studies were conducted per manufacturer’s instruction. Briefly, 50 µL of whole blood (WB) was inverted several times to mix RBC and plasma and added to 5 mL (1:100) of iso-osmolar polyvinylpyrrolidone (PVP) solution (pH 7.4, 37 °C) (R & R Mechatronics, Hoorn, Netherlands). Then the PVP + WB solution was loaded in the cup of the instrument. Measurements were performed at 37 °C with a camera gain of 208. The instrument software (Lorcca Maxsis software, version 5.08) generated data for RBCD curves of elongation indices (EI ) across the shear stress gradient, and two derived parameters: maximum elongation index (EI max) and the shear stress required to achieve half maximal deformability: SS_1/2_. Reduced RBCD was indicated by lower RBCD curves, lower EIs and EImax, and higher SS_1/2_. Given that SS_1/2_ is a derivative of all other shear stress gradient measures and a single continuous shear stress gradient measure most amenable to analysis, SS_1/2_ was used as the primary RBCD measure for exploratory analyses investigating associations with diabetes-related outcomes.

### Osmotic gradient ektacytometry

The same instrument was used as shear stress gradient ektacytometry, but with a different technique that varied osmolality (ranges from 50–500 osom at 37 C, camera gain 208) under constant shear stress of 30 Pa [14, 15, 23]. Per manufacturer‘s instruction, 250µL of WB blood was mixed with 5 mL iso-osmolar PVP solution and loaded into the instrument. The osmotic gradient parameters included the maximum elongation index obtained (O.EImax); the osmolality at which O.EImax is obtained (Omax); Omin, the osmolarity at the elongation index reaches a minimum (O.EImin) in the hypotonic arm of the curve; Ohyper: the osmolality at which the elongation index equals half of the normal maximum on the hypertonic arm of the curve (O.EIhyper). Of note, the elongation indices derived from osmotic gradient measures are characterized as “O.EIs”, to distinguish from shear stress gradient elongation indices (EIs).

Osmotic gradient ektacytometry provides information about cell water content, surface area to volume ratio (S/V ratio) and other parameters extensively discussed in elsewhere [13, 23]. The parameters pertinent to this study’s objectives are summarized as follows: Osmotic elongation indices (OEIs), particularly decreased OEImax in combination with other abnormal parameters, may indicate inherent structural membrane abnormality (such as alpha- or beta-spectrin defects in hereditary spherocytosis) [23]. Omin, obtained in hypotonic conditions provides information on both S/V ratio and hydration status, but also corresponds to the osmolality at which 50% of cells hemolyze, an equivalent measure of osmotic fragility [23]. RBCs with reduced S/V ratio (e.g. conditions with structural membrane defects such as congenital spherocytosis), will hemolyze at less hypotonic conditions (higher osmolality), thus higher Omin (increased osmotic fragility) [23]. Conversely, severely dehydrated RBCs (as in sickle cell disease), will reach their critical hemolytic volumes at more hypotonic conditions (lower osmolality), thus lower Omin (decreased osmotic fragility) [23]. Under hypertonic conditions, dehydrated and more viscous RBCs will reach their hypertonic elongation index (OEIhyper) at comparatively lower Ohyper values. Reduced Omax, in combination with reduced Omin and Ohyper (“left shift”) would indicate increased RBC dehydration that maybe independent of other concurrent abnormalities [23, 25]. 

### Osmotic fragility (NaCl 50% hemolysis)

Osmotic fragility studies were performed as described [26] with modifications. 150µL of varying concentrations of NaCl solution ranging from 0 to 0.9 g/100mL were added to 12 wells on a 96-well round bottom plate. Then 10µL of WB was added to all 12 wells. To avoid mechanical hemolysis, every well was gently mixed by pipetting three times. Test plates were incubated for 60 min at room temperature (~ 23 °C), followed by centrifugation at 1740 x g for 5 min at 4 °C. The supernatant was transferred into a new 96-well flat bottom plate and hemoglobin content was determined spectrophotometrically at λmax = 540 nm with a µQuant™ Microplate Spectrophotometer (Bio-Tek Instruments, Inc) using supernatant from the 0.90 g/100mL NaCl well as the blank. The well containing 0 g/100mL NaCl solution was used as 100% hemolysis control. The percent of hemolysis was calculated using the following formula: % Hemolysis = (Optical Density (O.D.) of test well − O.D. of 0.90 g/100mL NaCl well) ÷ (O.D. of dH2O well − O.D. of 0.90 g/100mL NaCl well). RBCs with increased osmotic fragility will hemolyze at higher NaCl concentrations (less hypotonic conditions) and will therefore have higher 50% NaCl hemolysis values. Conversely cells with decreased osmotic fragility will hemolyze at lower NaCl concentrations (more hypotonic conditions) and will therefore have lower 50% NaCl hemolysis values [26]. 

### Hemoglobin-oxygen dissociation rate (p50)

The p50 value (pO_2_ at which 50% of hemoglobin is saturated with O_2_), parameter of the hemoglobin − oxygen dissociation rate, was determined by using a HEMOX Analyzer (TCS Scientific Co., New Hope, PA). Oxygen dissociation curves were generated using dual wavelength spectrophotometry as described [27]. Briefly, human WB (50 µL) heparinized collection tube was diluted in 5 mL of Hemox-solution (HS-500, pH 7.4 ± 0.01, TCS Scientific Co.) mixed with 10 µL anti-foam agent (AFA-25, TCS Scientific Co.) and 20 µL bovine serum albumin solution (BSA-20, TCS Scientific Co.). The resulting mixture was gently mixed and heated to 37 °C, and then oxygenated to 100% under air purging. Samples were subsequently deoxygenated under continuous nitrogen purging. The p50 values were determined at the points of 50% oxygen saturation. Higher p50 values indicate increased hemoglobin-oxygen dissociation, while lower p50 values indicate decreased hemoglobin-oxygen dissociation.

### Statistical methods

The statistical methods are summarized below, with additional details and rationale provided in the Supplementary Methods. In this cross-sectional study, descriptive statistics were calculated for baseline characteristics of study participants. Counts and proportions were used for categorical variables, accompanied by Pearson’s chi-square test or Fisher’s exact tests as indicated by expected cell counts, while mean and standard deviations were used for continuous variables, accompanied by Welch’s t-test or Wilcoxon-Mann-Whitney tests as indicated by separate assessments of approximate normality.

The primary outcome investigating differences in cumulative elongation indices across a gradient of applied shear stresses (RBCD curves) in the diabetes cohort vs. nondiabetic controls was assessed using marginal models for repeated-measures-across-shear-stress-levels vectors (with each entry having their own Box-Cox-transformed values), fitted via generalized estimating equations with a working independence correlation model and empirical (sandwich-estimator) based standard errors to yield Wald-type test statistics. Given that the untransformed EI scale may not immediately convey the model-estimated absolute shift in the mean Box-Cox-transformed EI values, an inset plot shows such for diabetic and control cohorts relative to a ‘pooled’ mean of zero difference assumed under the null (see Supplementary Methods for additional detail) [28, 29]. To further explore that the primary study readout– the differences in RBCD curves in both groups– was not subject to bias due to group differences in baseline demographic/clinical covariates or covariates found to be significantly associated with associated with RBCD, we employed Wald-type statistics as done above, albeit with further one-predictor-at-a-time adjustment (see Supplementary Methods for additional details).

The secondary analyses of primary outcomes evaluating differences (estimated shifts) in mean elongation indices at each of the nine individual shear stress values and derived RBCD measures (maximal elongation index [EImax] and half-maximal shear stress [SS_1/2_]) in both groups were assessed using GEE-fitted marginal models adjusting for diabetes status and demographic characteristics. Exploratory analyses reported p-values for groups of related variables’ respective associations with SS_1/2_ via Wald-type test statistics from GEE-fitted marginal models adjusting for diabetes status and demographic characteristics (detailed explanations and rationales are provided in Supplementary Methods).

To determine how the cross-sectional study’s outcomes compare in study cohorts, graphically apparent trends in distributions are statistically tested across SS_1/2_ tertile subgroups within the diabetes cohort and nondiabetic controls using marginal models for repeated-measures-bivariate-outcome vectors (each unique pairing of SS_1/2_ with either Omin or p50, with each entry having its own Box-Cox-transformed values), fitted via generalized estimating equations with working exchangeable correlation models and empirical (sandwich-estimator) based standard errors to yield Wald-type test statistics (additional details provided in Supplementary Methods) [30]. Note that all analyses were conducted assuming: 5% significance; missing values were assumed to be missing at random, using available-case analyses; and using R version 4.0 or higher (www.r-project.org, see Supplementary Methods for full list of R packages employed, cited alongside each chosen method’s rationale).

## Results

### Enrollment flow of participants with and without diabetes

**Fig. 1:**
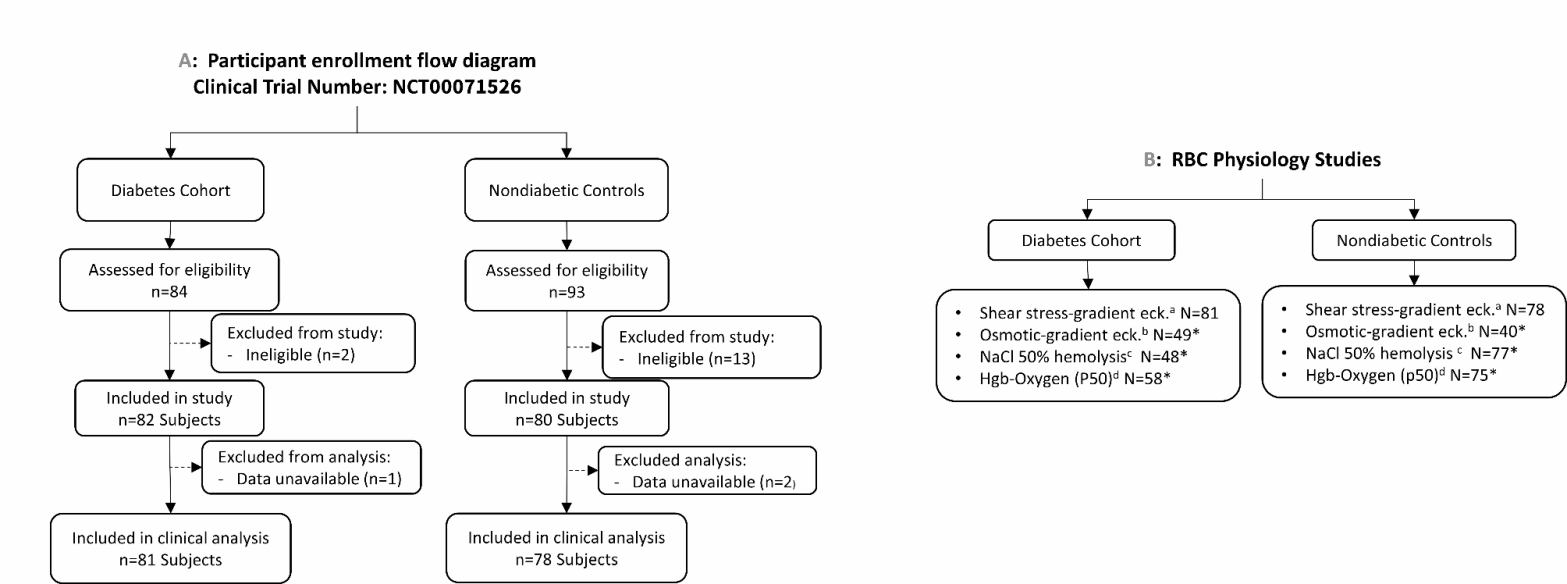
**A** Enrollment and patient flow of study participants in the diabetes cohort and nondiabetic control group, **B** Flow diagram for RBC physiology studies conducted in samples obtained from participants both groups. ^a^ Shear stress-gradient ektacytometry. ^b^ Osmotic-gradient ektacytometry. ^c^ Sodium chloride (NaCl) technique for osmotic fragility. ^d^ Hemoglobin-oxygen dissociation rate assessed by partial pressure at 50% oxygen saturation (p50)

A total of 177 individuals were assessed for eligibility for both study groups. Among the diabetes cohort, 84 participants were assessed for eligibility, 3 were excluded due to ineligibility or non-available data, 81 participants with diabetes were included in final clinical analysis (Fig. 1A). Among the 93 nondiabetic controls assessed, 15 were excluded and 78 nondiabetic participants were included in final clinical analysis (Fig. 1A). Samples from participants were used for shear stress-gradient ektacytometry studies (81 in diabetes cohort, and 78 nondiabetic controls), osmotic-gradient ektacytometry (49 in diabetes cohort and 40 nondiabetic controls), NaCl osmotic fragility studies (48 in diabetes cohort and 77 nondiabetic controls) and hemoglobin-oxygen dissociation studies (58 in diabetes cohort and 75 nondiabetic controls), Fig. 1B.

### Baseline characteristics of participants with and without diabetes

The demographic, clinical and laboratory characteristics of participants in both groups are shown in Table 1. Among the demographic covariates, the race/ethnic and sex composition in both groups were not significantly different, however the mean age was significantly higher in the diabetes cohort vs. nondiabetic controls (49 vs. 34 years, *p* < 0.05, Table 1). Among the baseline clinical parameters, the diabetes cohort had higher BMI (33 vs. 27 Kg/m^2^, *p* < 0.05), fasting glucose (146 vs. 88 mg/dL, *p* < 0.05) hemoglobin A1c (7.9 vs. 5.4%, *p* < 0.05), protein-to-creatinine ratio [PCR](0.270 vs. 0.096 mg/mg, *p* > 0.05),lower mean corpuscular volume [MCV] (86.2 vs. 87.9fL, *p* < 0.05) and lower eGFR (101 vs. 113 ml/min/1.73^2^, *p* < 0.05), Table 1. Among diabetes cohort, 75% had Type 2 and 25% Type 1, 24% had micro or macrovascular complications, mean duration of diabetes was 12 years (Table 1).

**Table 1:** Baseline demographic and clinical characteristics of participants in the diabetes cohort (*N* = 81) and nondiabetic controls (*N* = 78)

		Diabetes cohort	Nondiabetic controls
		*N* = 81	*N* = 78
Demographics: Mean (SD) or n (%)			
Race/Ethnicity^1^ n(%)			
White/Caucasian		33 (41%)	34 (44%)
Black/African American		36 (44%)	35 (45%)
Other^1^		12 (15%)	9 (11%)
Sex n(%)			
Male		39 (48%)	30 (38%)
Female		42 (52%)	48 (62%)
Age (years), Mean (SD)		49 (12)*	34 (13)*
Clinical and Lab Variables			
BMI (Kg/m^2^) – mean (SD)		32.5 (6.8)*	27.1 (5.3)*
Glucose (mg/dl) – mean (SD)		146 (53)*	88.4 (8.9)*
Hemoglobin A1C (%)* – mean (SD)		7.9 (1.5)*	5.4 (0.4)*
Hemoglobin (g/dl) – mean (SD)		12.9 (1.5)	14.3 (1.22)
Hematocrit (%) – mean (SD)		39.8 (3.5)	38.9 (3.8)
MCHC (g/dl) – mean (SD)		33.1 (1.2)	33.2 (1.1)
MCV (fl.) – mean (SD)		86.2 (5.1)*	87.9 (4.8)*
RBC count (10^6^ cells/µL) – mean (SD)		4.5 (0.43)	4.5 (0.45)
RDW (%) – mean (SD)		12.98 (0.9)	13.4 (1.13)
Systolic blood pressure (mmHg) – mean (SD)		129 (15)	120 (14)
Diastolic blood pressure (mmHg) – mean (SD)		75 (10)	70 (9)
eGFR^2^ (mL/min/1.73m^2^) – mean (SD)		101 (26)*	113 (21)*
PCR (mg/mg)* – mean (SD)		0.270 (0.56)*	0.096 (0.09)*
Total Cholesterol – Mean (SD)		173 (38)	171 (39)
HDL (mg/dl) – Mean (SD)		55 (17)	59 (16)
LDL (mg/dl) – Mean (SD)		94 (34)	93 (37)
Micro or Macrovascular disease – n (%)		20 (24%)	
Duration of Diagnosis (years) – mean (SD)		12 (11)	
Diabetes Type – n(%)			
Type 1		20 (25%)	
Type 2		61 (75%)	

BMI, body mass index; HDL, high density lipoprotein; LDL, Low density lipoprotein; MCHC, mean corpuscular hemoglobin concentration; MCV, Mean Corpuscular Volume; RDW, Red cell Distribution Width; PCR, protein creatinine ratio.

* Variables with statistically different values (p-Value < 0.05, Diabetes cohort vs. Nondiabetic controls, Welch’s *t-*test).

^1^ Race/ethnic categories were based on NIH reporting guidelines on racial and ethnic categories (NOT-OD-15-089). “Other” is composed of Asians, Hispanic and Latino and multiethnic subjects.

^2^ eGFR for all group was calculated using the Modification of Diet in Renal Disease v4 (MDRD4) equation: [186 x (Creatinine/88.4)-1.154 x (Age)-0.203 × (0.742 if female) x (1.210 if black)].

### Shear stress gradient RBC deformability (RBCD) measures in participants with versus without diabetes

**Fig. 2:**
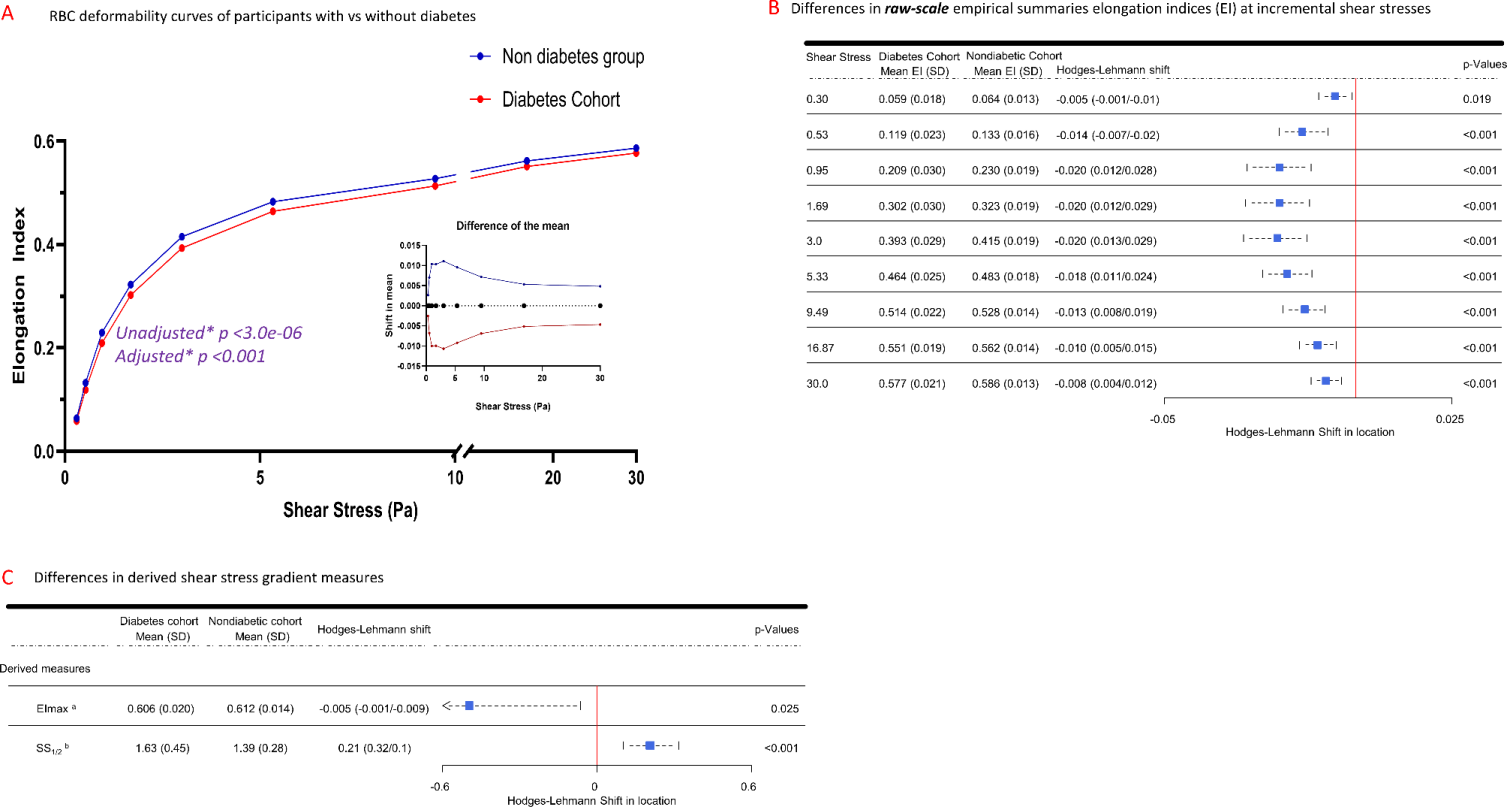
Shear stress gradient RBC deformability (RBCD) measures in the diabetes cohort (*N* = 81) and nondiabetic controls (*N* = 78). **A** RBC deformability curves of mean elongation indices across a gradient of shear stresses (0.3–30 Pa) in the diabetes cohort and nondiabetic controls. The difference of the mean values in both groups is shown in the insert; p-values reflect joint Wald tests for diabetes-diagnosis effects of repeated measures modeling across shear-stress levels using transformed values. **B** Mean elongation index at each individual shear stress value in both groups. Squares indicate Hodges-Lehmann shifts, with horizontal points indicating 95% Confidence Interval (CI). **C** Derived RBCD shear stress-gradient measures: maximal elongation index: EImax^a^, and shear stress required to reach 50% of EImax: SS_1/2_^b^. Squares indicate Hodges-Lehmann shifts, with horizontal points indicating 95% CI. *Adjusted for potentially confounding covariates (Supplementary Fig. 1). ^a^ Maximum elongation index (EImax). ^b^ Shear stress value required to reach half-maximal elongation index (SS_1/2_)

Characterization and assessment of RBCD parameters in diabetes were based on shear stress gradient ektacytometry [1, 2, 4]. Red blood cell deformability (RBCD) curves of the mean elongation indices (EI) across the nine incremental shear stresses ranging from 0.30 to 30 Pa are shown for the diabetes cohorts and nondiabetic controls (Fig. 2A). The RBCD curve for the diabetes cohort was significantly different (lower) compared with the nondiabetic controls (*p* < 0.001, Fig. 2A). The largest differences in mean EI values were observed at shear stress of 0.95, 1.69 and 3.0 pascals (Fig. 2A insert, Supplementary Table 1A). Differences in the RBCD curves remained significant following adjustments for potential confounding demographic and clinical covariates (Fig. 2A, Supplementary Table 1B).

To further characterize RBCD differences at each individual shear stress, we compared the EIs at all nine individual shear stresses and found significantly lower EIs in the diabetes cohort vs. nondiabetic controls (p-values < 0.001 for all SS values except 0.3pa [p-value = 0.019], Fig. 2B), with the biggest differences at shear stress values of 0.95, 1.69 and 3.0 Pa, recapitulating similar findings (Fig. 2A inset). Among the derived RBCD measures, the diabetes cohort had significantly lower maximum EI [EImax] (0.606 ± 0.02 vs. 06.12 ± 0.014, *p* = 0.025) and higher shear stress required to reach half of EImax [SS_1/2_] (1.63 ± 0.45 vs. 1.39 ± 0.28, *p* < 0.001), compared with the nondiabetic controls (Fig. 2C). These data indicate that all shear stress gradient RBCD parameters were substantially different in the diabetes cohort vs. nondiabetic controls, with biggest differences observed at lower shear stress values. SS_1/2_ was used as a primary continuous measure of RBCD for subsequent analyses.

### Demographic and clinical parameters associated with reduced RBCD (higher SS_1/2_), in all participants [adjusted for diabetes status and demographic differences]

**Fig. 3:**
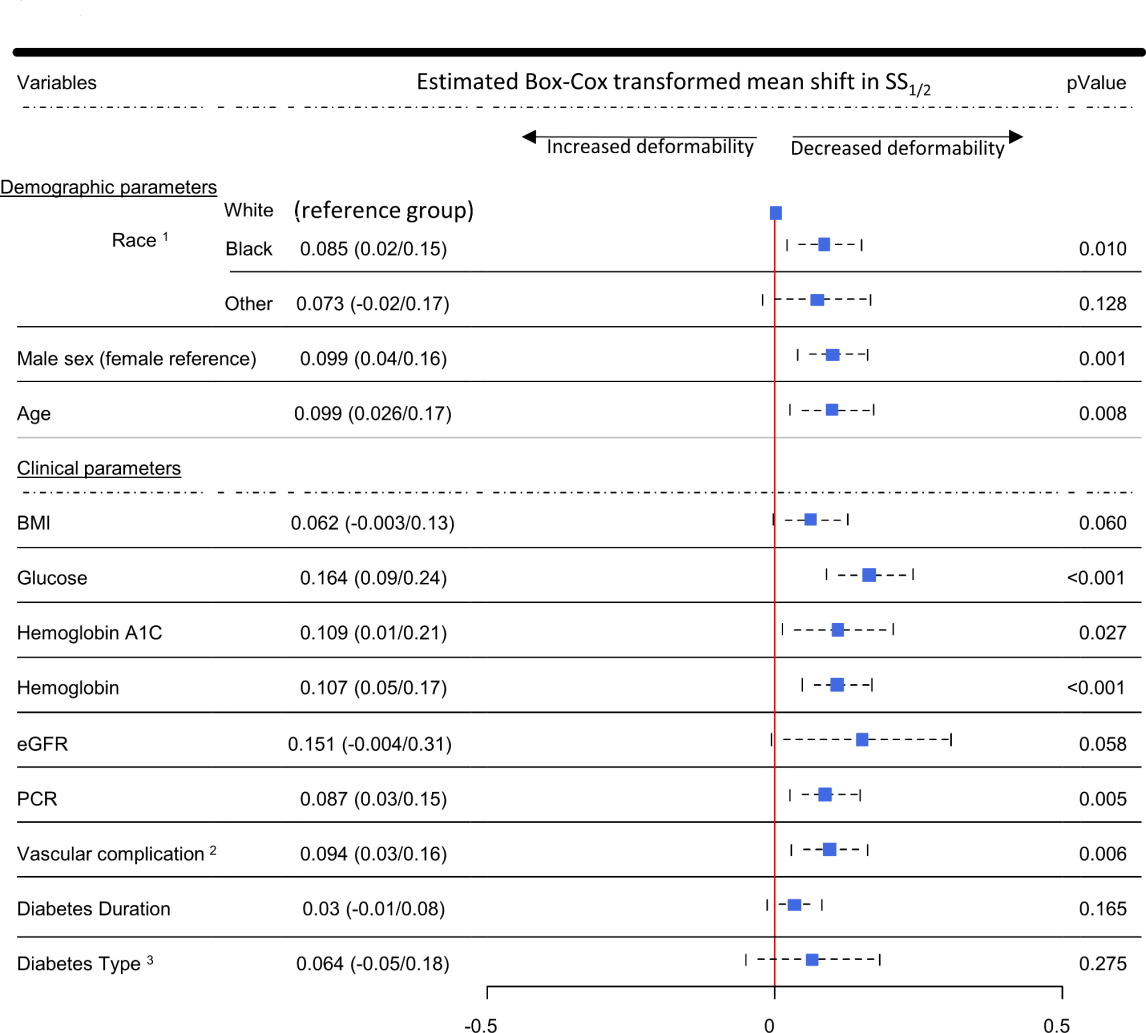
Demographic and clinical variables and their predictive association with RBCD assessed using diabetes-status-adjusted linear regression of transformed values for SS_1/2_. Squares indicate Box-Cox transformed mean shifts in SS_1/2_, conditional on diabetes status, with horizontal points indicating 95% CI. ^1^Black and White groups were comprised of non-Hispanic participants. “Other” was comprised of Asians, Hispanic/Latino ethnicity, and multiracial participants. ^2^Presence of micro- or macrovascular complications. ^3^Type 1 or Type 2 diabetes. BMI, body mass index; eGFR, estimated glomerular filtration rate; PCR, Protein-to-creatinine ratio

Among the demographic variables, Black race [relative to White race] (*p* = 0.01), male sex [relative to female sex] (*p* = 0.001) and older age (*p* = 0.008) were associated with increased SS_1/2_ (decreased RBCD). Among clinical parameters assessed, covariates associated with decreased RBCD included higher fasting plasma glucose (*p* < 0.001), hemoglobin concentration (*p* < 0.001), hemoglobin A1c (*p* = 0.027), increased protein-to-creatinine (PCR) ratio (*p* = 0.005) and presence of micro/macrovascular complications (*p* < 0.006, Fig. 3). The association with Body mass index (BMI), renal function (estimated glomerular filtration rate), diabetes type and duration did not reach statistical significance (Fig. 3).

### Osmotic gradient ektacytometry studies in the diabetes cohort versus nondiabetic controls

**Fig. 4:**
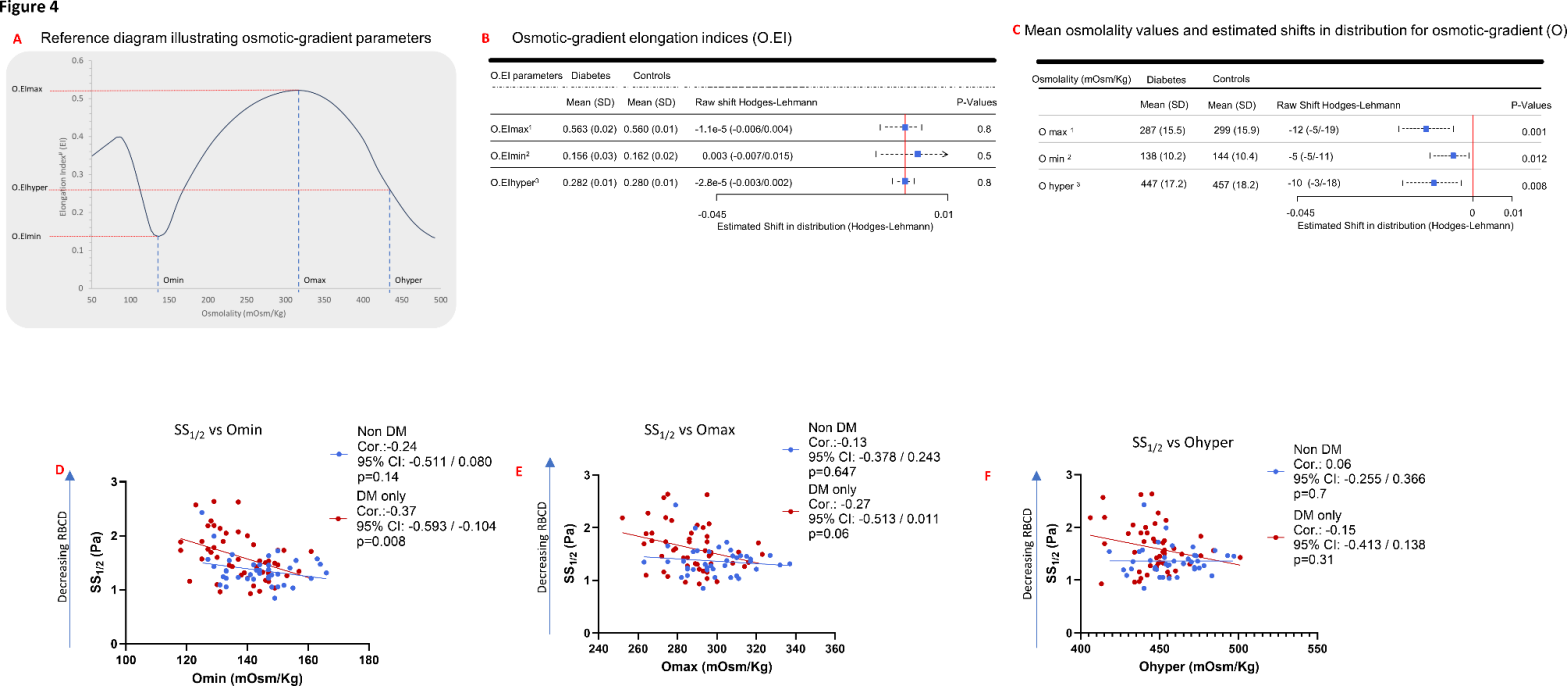
Relationships between RBC deformability (SS_1/2_) and osmotic-gradient osmolality measures (Omax, Omin, Ohyper) in the diabetes cohort (*N* = 49) and nondiabetic controls (*N* = 40). A Representative scheme of osmotic gradient curves and parameters. (**B**-**C**) Mean osmotic elongation indices (**B**), and corresponding mean osmolality values (**C**) in both groups. Squares indicate Hodges-Lehmann shifts, with horizontal points indicating 95% CI. (**D**-**F**) Associations between RBCD (SS_1/2_) and osmotic-gradient osmolality measures: Omin (**D**), Omax (**E**) and Ohyper (**F**) within the diabetes cohort (red circles) and within nondiabetic controls (blue circles) ^1^Maximum osmotic elongation index (O.EImax) and corresponding osmolality (Omax) ^2^Minimum osmotic elongation index (O.EImin) and corresponding osmolality (Omin) ^3^Hypertonic osmotic elongation index (O.EIhyper) and corresponding osmolality (Ohyper)

Osmotic gradient ektacytometry studies were conducted to investigate the RBC changes associated with RBCD in both groups. Abnormal RBCD could potentially result from: abnormal structural membrane abnormalities, resulting in reduced surface area-to-volume ratio (S/V ratio); impaired RBC volume status resulting in increased intracellular viscosity; or a combination of both factors [23]. The osmotic gradient studies were used to investigate which of these three processes might be impacted in diabetes. A reference diagram illustrating the osmotic-gradient parameters is shown in Fig. 4A (see Methods section for detailed definitions and descriptions of osmotic gradient parameters). The osmotic elongation indices were not different, with maximum osmolality [O.EImax] (0.563 ± 0.02 vs. 0.560 ± 0.01, *p* = 0.8), minimum osmolality [O.EImin] (0.156 ± 0.03 vs. 0.162 ± 0.02, *p* = 0.5) and hypertonic osmolality [O.EIhyper] (0.282 ± 0.01 vs. 0.280 ± 0.01, *p* = 0.8) Fig. 4B. In contrast, all three osmolality values were significantly reduced in the diabetes cohort vs. nondiabetic controls, with lower minimum osmolality [Omin] (138 ± 10.2 vs. 144 ± 10.4, *p* = 0.012), maximum osmolality [Omax] (287 ± 15.5 vs. 299 ± 15.9, *p* = 0.001) and hypertonic osmolality [Ohyper], (447 ± 17.2 vs. 457 ± 18.2, *p* = 0.008), Fig. 4C. Based on the significant differences in osmolality parameters between both groups (Fig. 4C), subsequent osmotic-gradient analyses were focused on osmolality measures.

### Relationships between RBCD (SS1/2) and osmotic gradient measures in both cohorts

In the diabetes cohort, higher SS_1/2_ (reduced RBCD) was significantly associated with lower Omin (Corr. −0.37, 95% CI: −0.593 to −0.104, *p* = 0.008), but not Omax (Corr. −0.27, 95% CI: −0.513 to 0.011, *p* = 0.06) or Ohyper (Corr. −0.15, 95% CI: −0.413 to 0.138, *p* = 0.31), Fig. 4D-F. No significant associations were observed within the nondiabetic control group for Omin (Corr. −0.24, 95% CI: −0.511 to 0.080, *p* = 0.14), Omax (Corr. −0.13, 95% CI: −0.378 to 0.243, *p* = 0.65) or Ohyper (Corr.0.06, 95% CI: −0.255 to 0.366, *p* = 0.7), Fig. 4D–F.

### Relationship between osmotic fragility (omin) and RBCD (SS1/2) in both groups

**Fig. 5:**
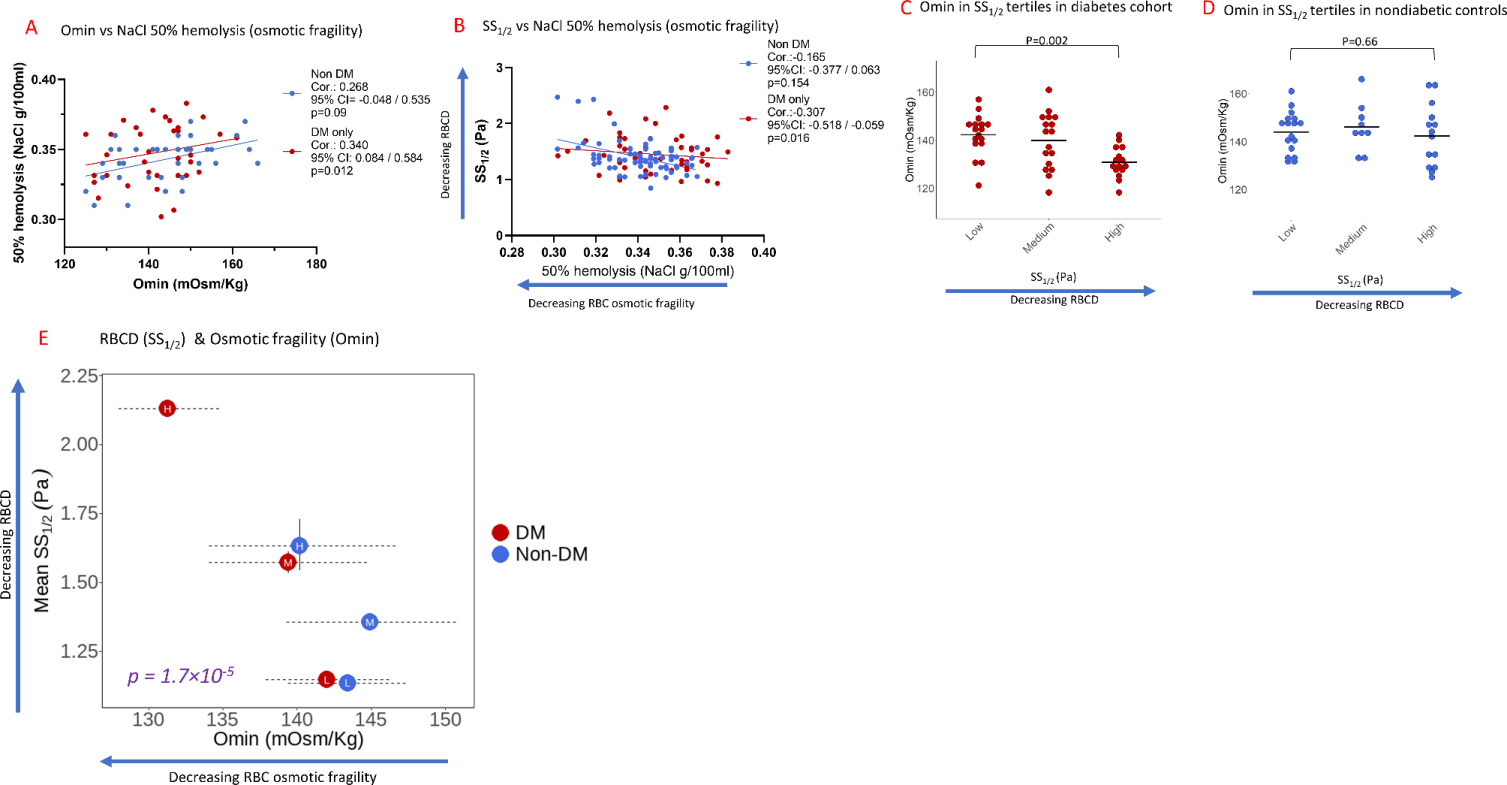
RBC Deformability (SS_1/2_) and Osmotic fragility (Omin) in the diabetes cohort (*N* = 48) and nondiabetic controls (*N* = 77). **A** Associations between Omin and osmotic fragility measured using the classic NaCl technique (See Methods) in the diabetes cohort (red circles) and nondiabetic controls (blue circles); p-values reflect joint Wald tests for diabetes-diagnosis effects of repeated measures modeling across SS_1/2_ tertiles using transformed values. **B** Associations between SS_1/2_ and NaCl osmotic fragility the diabetes cohort (red circles) and nondiabetic controls (blue circles). (C-D) Mean Omin values stratified by SS_1/2_ tertiles within the diabetes cohort (**C**) and within nondiabetic controls (**D**). **E** Relationships between RBCD (SS_1/2_) and mean osmotic fragility (Omin) in subgroups stratified by SS_1/2_ tertiles within the diabetes cohort (red circles) and nondiabetic controls (blue circles); 95% confidence intervals result from repeated measures modeling across SS_1/2_ tertiles using transformed values. SS_1/2_ tertiles within the diabetes cohort: low: 0.75–1.37 Pa; medium:1.38–1.75; high:1.76–2.9 Pa, and nondiabetic controls: low:0.85–1.28 Pa; medium:1.29–1.41 Pa; high: 1.43–2.47 Pa

Given that Omin showed the strongest associations with RBCD (Fig. 4), subsequent studies were focused on characterizing Omin in our study cohorts. First, to validate Omin as a measure of osmotic fragility, Omin was compared with the commonly used technique for osmotic fragility: NaCl 50% hemolysis (Fig. 5A, see Methods for details). Omin was significantly correlated with NaCl osmotic fragility within the diabetes cohort (Corr. = 0.34, 95% CI: 0.084 to 0.584, *p* = 0.012), but not within the nondiabetic controls (Corr.= 0.27, 95% CI: −0.048 to 0.535, *p* = 0.09), Fig. 5A. Additionally, NaCl 50% studies recapitulated similar associations observed with Omin (Fig. 4D), with significant association between NaCl 50% hemolysis and SS_1/2_ in the diabetes cohort (Corr.= −0.31, 95% CI: −0.518 to −0.059, *p* = 0.016), but not the nondiabetic controls (Corr.= −0.17, 95% CI: −0.377 to 0.063, *p* = 0.15), Fig. 5B. Findings validated Omin as a measure of osmotic fragility in a diabetes cohort.

To expand on these findings, we investigated Omin differences in subgroups stratified by SS_1/2_ tertiles (see Methods for detailed description) within the diabetes cohort (Fig. 5C) and within the nondiabetic controls (Fig. 5D). Within the diabetes cohort, participants in the high-tertile subgroup had significantly lower mean Omin vs. participants in the low-tertile subgroup (131 ± 6.2 vs. 142 ± 8.5 mosm/kg, *p* = 0.002, Fig. 5C, Supplementary Table 2A), however no such difference was observed within the nondiabetic controls (142 ± 12.5 vs. 144 ± 8.5 mosm/kg, *p* = 0.66, Fig. 5D, Supplementary Table 2A). Across all subgroups of both the diabetes cohort and nondiabetic controls, higher SS_1/2_ (reduced RBCD) was significantly associated with lower Omin [reduced osmotic fragility] (*p* < 0.001, Fig. 5E) with high-tertile diabetes subgroup showing the highest SS_1/2_ and lowest Omin (Fig. 5E). Findings indicate that the relationship between RBCD and osmotic fragility varies within the diabetes cohort, but not the diabetic control group.

### Relationships between RBCD (SS1/2) and functional RBC measure: hemoglobin-oxygen dissociation rate (p50) in both groups

**Fig. 6:**
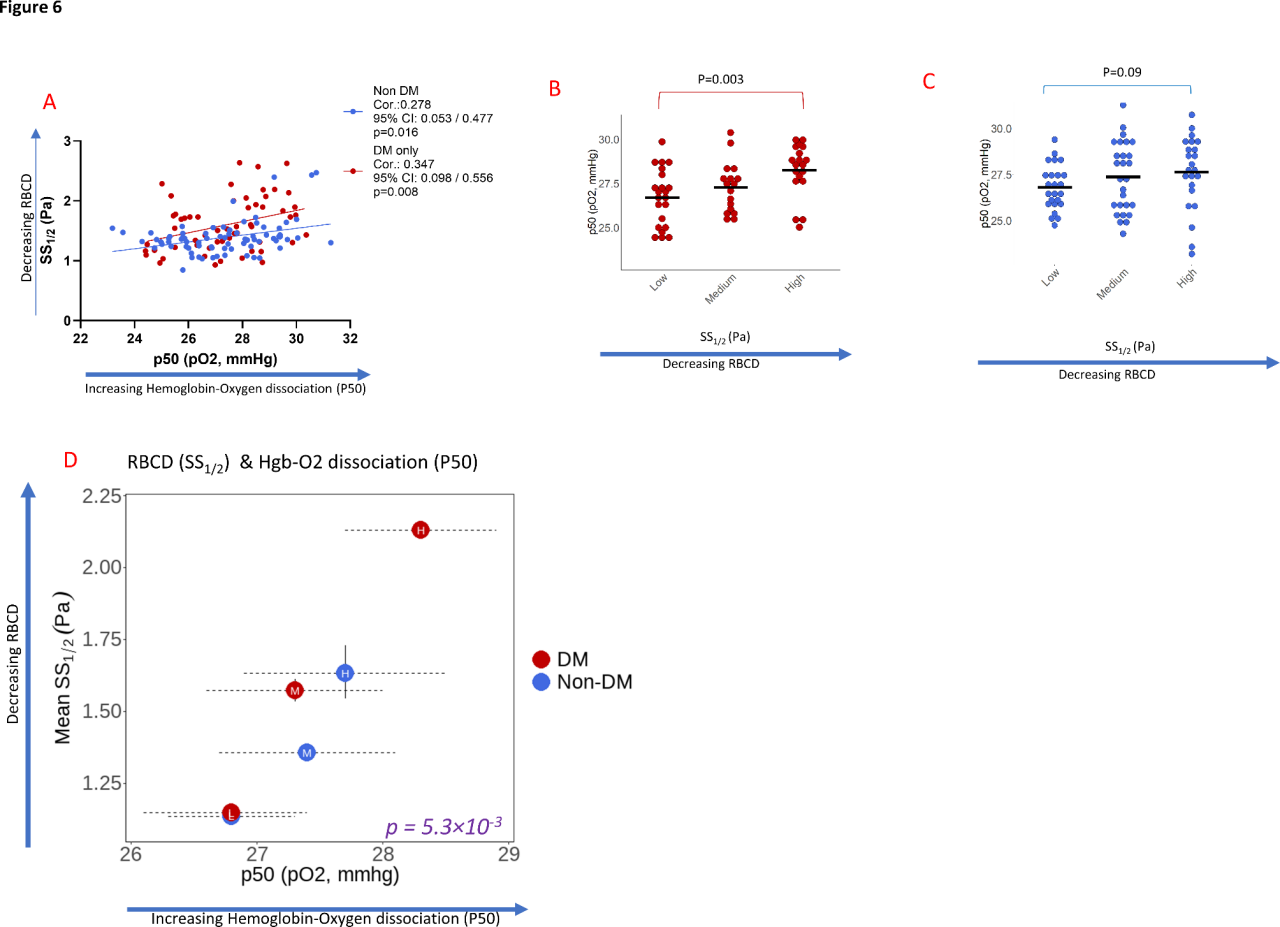
RBC Deformability (SS_1/2_) and hemoglobin-oxygen dissociation rate (p50) in the diabetes cohort (*N* = 58) and nondiabetic controls (*N* = 75). **A** Associations between RBCD (SS_1/2_) and hemoglobin-oxygen dissociation rate (p50) within diabetes cohort (red circles) and nondiabetic controls (blue circles); p-values reflect joint Wald tests for diabetes-diagnosis effects of repeated measures modeling across SS_1/2_ tertiles using transformed values. **B**, **C** Mean p50 values in subgroups stratified by SS_1/2_ tertiles within the diabetes cohort (**B**) and nondiabetic controls (**C**). **D** Relationships between RBCD (SS_1/2_) and mean p50 values in subgroups stratified by SS_1/2_ tertiles within the diabetes cohort (red circles) and nondiabetic controls (blue circles); 95% confidence intervals result from repeated measures modeling across SS_1/2_ tertiles using transformed values. SS_1/2_ tertiles within the diabetes cohort: low: 0.75–1.37 Pa; medium:1.38–1.75; high:1.76–2.9 Pa, and nondiabetic controls: low:0.85–1.28 Pa; medium:1.29–1.41 Pa; high: 1.43–2.47 Pa

We investigated how RBCD might impact RBC function: hemoglobin-oxygen dissociation (p50). There was a significant association between higher SS_1/2_ (decreased RBCD) and higher p50 values (increased hemoglobin-oxygen dissociation) within the diabetes cohort (Corr.=0.35, 95% CI: 0.098 to 0.556, *p* = 0.008) and within the nondiabetic controls (Corr.= 0.28, 95% CI: 0.053 to 0.477, *p* = 0.016), Fig. 6A. We investigated differences in p50 values in subgroups of SS_1/2_ tertiles within the diabetes cohort (Fig. 6B) and within the nondiabetic controls (Fig. 6C). Within the diabetes cohort, participants in the high-tertile subgroup had significantly higher mean p50 vs. participants in the low-tertile subgroup (28.3 ± 1.5 vs. 26.7 ± 1.6 mmHg, *p* = 0.003, Fig. 6B, Supplementary Table 2B), however no difference was observed within the nondiabetic controls (27.7 ± 2.0 vs. 26.9 ± 1.2 mmHg, *p* = 0.09, Fig. 6C, Supplementary Table 2B). Across all subgroups in the diabetes cohort and nondiabetic controls, higher SS_1/2_ (reduced RBCD) was significantly associated with higher hemoglobin-oxygen dissociation rate (*p* < 0.01, Fig. 6D) with high-tertile diabetes subgroup showing the highest SS_1/2_ and hemoglobin-oxygen dissociation rate (Fig. 6D). Findings are consistent with a structure-function relationship, with significant variation within the diabetes cohort, but not the nondiabetic controls.

## Discussion

In this study, we characterized red blood cell deformability (RBCD) parameters pertinent to diabetes, predictive demographic and clinical covariates, and diabetes-specific RBC changes associated with altered RBCD. The diabetes cohort had significantly reduced RBCD, even after adjustments for potential confounding demographic and clinical variables that were either different at baseline (Table 1) or independently associated with reduced RBCD (Fig. 3, Supplementary Table 2). Reduced RBCD was strongly associated with aberrant osmotic-gradient ektacytometry parameters suggestive of RBC dehydration in diabetes, with the most profound associations observed with reduced osmotic fragility (Figs. 4 and 5). A structure-function relationship was observed with reduced RBCD, reduced osmotic fragility and increased hemoglobin-oxygen dissociation (Figs. 5 and 6). Findings reveal pathophysiologic RBC changes in diabetes that are inconsistent with hypothesis linking reduced RBCD to increased RBC rigidity and increased osmotic fragility. However, findings provide strong evidence for disordered oxygen release as a functional consequence of reduced RBCD in diabetes [6, 16–18].

In characterizing RBCD in diabetes, we investigated whether specific ektacytometry measurement approaches might be more sensitive and applicable to a diabetes population. We focused only on shear stress gradient parameters—the basis for recent RBCD studies in diabetes and found significant differences in all raw and derived shear-stress gradient measures (Fig. 2) [1, 2, 4]. Given that several ektacytometry-based studies of populations with diabetes used shear stress values ranging from 1.13 Pa to 3pa [1, 2, 4, 31], we investigated differences across shear stresses using two separate analyses (Fig. 2A inset and Fig. 2B), and found the magnitude of between-group differences were highest at 0.95 Pa, 1.69 Pa and 3.0 Pa, validating use of lower shear stress values in investigating RBCD in diabetes [1, 2, 4, 31]. These data indicate that comparative rheological differences in diabetes vs. controls are more discernable at lower, rather than higher shear stresses. An explanation is that shear stress in the microvasculature is predominantly impacted by forces of viscosity rather than forces of high flow (inertia) [32]. Thus, increased viscosity from less deformable RBCs will require comparatively higher shear stresses to maintain flow/perfusion in the microcapillaries.

**Fig. 7:**
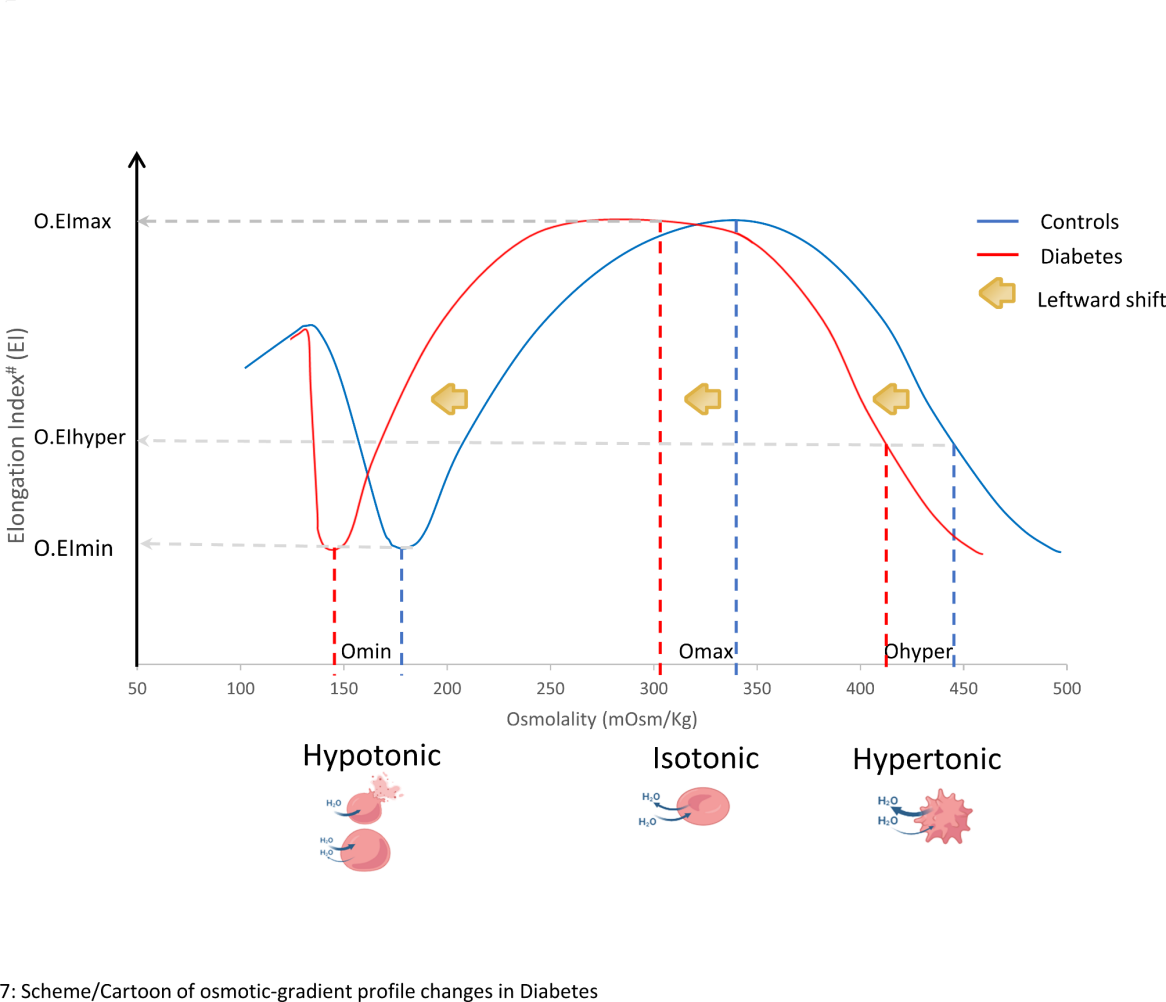
Scheme of osmotic-gradient profile changes in diabetes

In contrast to shear stress gradient ektacytometry in which RBCD indices are obtained over a spectrum of shear stresses and at fixed osmolality, osmotic gradient ektacytometry involves dynamic changes to osmolality (hypotonic, isotonic, and hypertonic conditions) at fixed shear stress values [14, 15, 23]. Osmotic gradient ektacytometry provides a powerful yet not previously described means to investigate RBC structural changes in diabetes. Our study showed significantly reduced osmolality measures (Omax, Omin and Ohyper) in the diabetes cohort vs. nondiabetic controls (Fig. 4), consistent with a “leftward shift” along the osmotic axis in the osmotic-gradient curve (Fig. 7: scheme), and indicative of disordered RBC dehydration [23]. While similar “leftward shift” has been described in hematological disorders like sickle cell disease and hereditary xerocytosis [23, 25], a distinguishing feature in the diabetes cohort was the similar maximum elongation index (O.EImax) relative to nondiabetic controls (Fig. 4), findings that suggest structural membrane abnormalities (e.g. defects in alpha or beta spectrin) are less likely a factor in diabetes [23]. While precise underlying mechanisms that lead to dysregulation in RBC hydration are unknown, several possibilities include glucose-mediated dysregulation of RBC membrane channels that regulate volume and cation flux, including Na/K ATPase pump, aquaporins, PIEZO1, K-Cl cotransport (KCC) and Gardos channel [18, 25, 33]. 

It was notable that while the maximum elongation indices derived using osmotic-gradient ektacytometry [O.EImax] were similar in the diabetes cohort and nondiabetic controls (Fig. 4B), the equivalent measure derived from shear stress ektacytometry (EImax) was significantly different in both groups (Fig. 2C). Analyses of shear stress-gradient measures only in participants who obtained osmotic-gradient studies, showed decreased EImax and increased SS_1/2,_ consistent with data from the full cohorts (Fig. 2C, Supplementary Fig. 1) that showed reduced RBCD in diabetes. Despite prior assumptions that osmotic gradient and shear stress gradient data provide similar information, our findings provide evidence that osmotic gradient measures may not be an appropriate or equivalent substitute for shear stress gradient measures in characterizing RBCD in diabetes, or in clinical studies evaluating cardiometabolic risk outcomes [34]. 

With an understanding of the osmotic gradient profile in diabetes, we investigated the relationship between RBCD (using SS_1/2_) and these aberrant osmolality measures. Among the osmolality measures, only Omin was significantly associated with reduced RBCD. While Omin values are said to correspond RBC osmotic fragility—the osmolality at which 50% of cells are hemolyzed [13, 23, 26], it was unknown whether this correlation would be applicable to a diabetes population, as studies establishing this correlation were based on modified cell studies unrelated to diabetes. In the diabetes cohort, we found significant association between Omin and osmotic fragility measured using the traditional approach[NaCl 50% hemolysis]^26^, (Fig. 5A, Methods). Taken together, these data recapitulate a relationship between reduced RBCD and reduced osmotic fragility (Omin), likely resulting from dysregulated RBC hydration status [23]. 

One potential consequence of reduced RBCD in diabetes and its predisposition to diabetic microvascular complications is impaired hemoglobin-oxygen delivery, a critical function of RBCs. Increased hemoglobin-oxygen dissociation (higher p50 values) suggests weaker hemoglobin affinity for oxygen, facilitating increased oxygen release to the tissues. Our data indicated a structure-function relationship between reduced RBCD (higher SS_1/2_), lower osmotic fragility (lower Omin) and higher hemoglobin-oxygen dissociation (higher P50), in subgroups within the diabetes cohort and nondiabetic controls subgroups stratified by SS_1/2_ tertiles (Figs. 5E and 6D). In contrast with more constrained subgroups of the nondiabetic controls, subgroups within the diabetes cohort were more divergent and distinct, with the high-tertile subgroup showing the most extreme values, and the low-tertile diabetic subgroup having similar values as the nondiabetic controls (Figs. 5E and 6D). Independent of RBCD, lower osmotic fragility (Omin) was significantly associated with higher p50 values in the diabetes cohort (*p* = 0.038, Supplementary Fig. 2A), recapitulating structure-function relationships.

While studies have described changes in p50 as a function of hyperglycemia, our data suggest p50-RBCD relationship may be independent of hyperglycemia for two reasons: First, p50-RBCD association was significant in the normoglycemic nondiabetic controls (Corr.0.28, *p* = 0.016), albeit with weaker correlation than the diabetes cohort (Corr.0.35, *p* = 0.008, Fig. 6A). Second, the significant difference in p50 values observed in high- vs. low-tertile diabetic subgroups (Fig. 6B) was not accompanied with differences in glycemic parameters (Supplementary Fig. 2B). Additionally, we found no significant correlation between p50 and either fasting glucose or hemoglobin A1c in the diabetes cohort (Supplementary Fig. 2C and 2D). These data imply that p50 changes may be more a consequence of the structural RBC changes such as osmotic fragility, rather than hyperglycemia. Long-term consequences of p50 changes and the predisposition to vascular complications in diabetes will require dissecting new mechanisms revealed here in combination with longitudinal clinical studies in diabetic subjects.

Our study revealed notable demographic and clinical associations with reduced RBCD, including older age, Black race, male sex, and hyperglycemia–all risk factors for diabetic vascular complications [35–40] (Fig. 3). Reduced RBCD was also associated with higher hemoglobin concentrations, likely due to physiologically higher hemoglobin concentrations in males–that is independent of diabetes status (Supplementary Fig. 3A). To further explore the how sex differences and diabetes status variably impact RBC physiology, we compared subgroup differences in mean corpuscular hemoglobin concentration (MCHC), a measure of RBC hydration and viscosity as evidenced by the significant association with Ohyper, an osmotic gradient measure of cellular hydration (Supplementary Fig. 3B) [15, 22, 23, 25]. Within the diabetes cohort, there was comparatively higher MCHC concentrations male vs. female participants, but not within the nondiabetic controls (Supplementary Fig. 3C). Findings suggest association between male sex and reduced RBCD (Fig. 3), may be a combination of pathologically higher MCHC in males with diabetes—perhaps from dehydrated RBCs, and physiologically higher hemoglobin concentrations in males independent of diabetes. These findings highlight the complex physiological and pathological factors that likely modulate RBCD changes independently of diabetes status. Beyond diabetes, findings may offer mechanistic insights in conditions such as RBC storage lesion, where pathophysiological RBC changes and donor-related factors potentially impacts storage quality and possibly RBC transfusion efficacy [41–43]. 

There are several strengths of the study presented here. This study included the first description of the demographic and clinical covariates predictive of reduced RBCD in diabetes, and the first known use of osmotic gradient ektacytometry to characterize structural RBC changes in diabetes, with mechanistic insights that point towards disordered RBC dehydration in diabetes. While cross-sectional study design provides a critical first step in evaluating relationships, limitations include the inability to characterize dynamic and longitudinal relationships between clinical parameters such as glycemic changes, and their effect on RBCD measures. Additionally, this study was not powered to investigate subgroup differences based on race and demographic parameters. The scope of this study did not include other potential factors implicated in RBCD, including genetics, chronic inflammation, oxidative stress [44, 45]. Another limitation is that not all participants obtained all RBC physiology studies (Fig. 1B) due to availability of supplies and/or instrument, however analysis for potential selection bias indicated no marked differences in demographic characteristics other than a single comparison (out of two dozen) that exceeded the ‘moderate’ or ‘medium’ levels (Supplementary Fig. 4) [46]. 

Lastly, the diabetes cohort was a relatively well controlled cohort with an average A1C of 7.9%, only 24% had microvascular complications and excluded smokers and individuals with known cardiovascular disease, limiting our understanding in populations with advanced complications such as coronary artery disease and chronic kidney diseases While it is possible our findings underestimate the scope of RBCD-related dysregulation in diabetes, this relatively well controlled diabetes cohort offers a mechanistic window in early pathophysiological changes that may precede overt clinical manifestations of diabetic vascular complications.

In conclusion, this study showed that aberrant RBCD in diabetes was independent of significant demographic and clinical covariates, with potentially early pathophysiological RBC changes, as assessed by shear stress ektacytometry, osmotic gradient ektacytometry, and p50. In addition to expanding our understanding of RBC pathophysiology in diabetes, these data provide a critical foundation for future studies aimed at understanding how RBCD measures can be utilized in the early recognition, mitigation, and management of vascular complications in diabetes. Comprehensive larger and longitudinal studies are needed to better understand mechanisms and clinical implications of early RBCD changes in diabetes.

## Electronic supplementary material

Below is the link to the electronic supplementary material.

Supplementary Material 1

Supplementary Material 2

## Data Availability

Data described in the manuscript will be made available upon request pending application and approval. No datasets were generated or analysed during the current study.

## Abbreviations

BMI: Body mass index
eGFR: Estimated glomerular filtration rate
EI: Elongation index
EImax: Maximal elongation index
LORRCA: Laser-assisted optical rotational red cell analyzer
MCHC: Mean corpuscular hemoglobin concentration
MCV: Mean corpuscular volume
O.EI: Osmotic elongation index
O.EIhyper: Osmotic-gradient hypertonic elongation index
O.EImax: Osmotic-gradient maximum elongation index
O.EImin: Osmotic-gradient minimum elongation index
Ohyper: Hypertonic osmolality
Omax: Maximum osmolality
Omin: Minimum osmolality
p50: Partial pressure of oxygen at 50% saturation of hemoglobin
PCR: protein creatinine ratio
pO2: Partial pressure of oxygen
RBC: Red blood cell
RBCD: Red blood cell deformability
RDW: Red blood cell distribution width
SS: Shear stress
SS_1/2_: Half-maximal shear stress
S/V: Surface area-to-volume ratio
WB: Whole blood
