# Supplementary material for: Altered RBC deformability in diabetes: clinical characteristics and RBC pathophysiology
Source: Cardiovasc Diabetol. 2024 Oct 18;23:370. doi: 10.1186/s12933-024-02453-2 (PMC11490132; doi:10.1186/s12933-024-02453-2)
Supplement: Supplementary file 1 — Supplementary Material 1 [file 12933_2024_2453_MOESM1_ESM.docx]

SUPPLEMENTARY STATISTICAL METHODS

The statistical methods briefly summarized in the main manuscript are detailed further below, organized by outcome subsections.

***Primary Outcome: Approach to Primary Analyses***

The primary outcome—investigating cumulative differences among elongation indices (EIs) across a gradient of applied shear stresses (RBCD curves, [Figure 2A](#_Figure_2A)) in the diabetes cohort vs nondiabetic controls—was assessed using marginal models for repeated-measures-across-shear-stress-levels vectors (with each entry having their own [Box-Cox](https://CRAN.R-project.org/package=DescTools)-transformed values), fitted via generalized estimating equations with a working independence correlation model and empirical (sandwich-estimator) based standard errors to yield Wald-type test statistics using the [geepack package in R](https://CRAN.R-project.org/package=geepack). As this entails repeated-measures modeling of a continuous outcome, GEE-based estimation for marginal mean inferences (like differences due to diabetes) is preferred in this case to other methods (e.g., mixed-effects repeated measures models) primarily because of the robustness of such inferences to assumptions about the interdependence among the repeated measures, i.e., the ‘working correlation’ model.

To assess robustness of the primary repeated-measures *marginal* model-based analysis finding, especially when considering other contributing (and potentially confounding) factors, we explored whether further ‘adjustment’ (one additional-predictor-at-a-time) changed inferences relative to the referent ‘unadjusted’ model with diabetes status. Each such model asserted the findings for overall RBCD curve (adjusted p-value range shown in purple font within [Figure 2A](#_Figure_2A) and are listed in Supplementary Tables).^1,2^ For these comparisons, we employed Wald-type statistics as done above, albeit adjusting simultaneously only for diabetes status; the rationale for using a select subset of features is described next. This select subset of covariates overlaps with those denoted in Table 1 for cohort comparison by asterisks (statistically significant differences by diabetes status per Pearson’s chi-square test of counts/proportions or categorical variables, and Welch’s t-test for continuous measures, each using [base R stats functions](https://cran.r-project.org/)) as well as any associated with ektacytometry-based summary measure of the overall curve, SS_1/2_, per methods outlined in descriptions below.

To provide more intuitive depiction of the modeled RBCD comparisons ([Figure 2A](#_Figure_2A) inset plot) we determined differences in absolute shift in the mean Box-Cox-transformed EI values between the diabetic and control cohorts relative to a ‘pooled’ mean of zero difference assumed under the null, each such difference as estimated by the marginal model for repeated measures (Supplementary Tables). The mean EI values across corresponding shear stresses were implicitly estimated in the combined cohort, with regression parameterization distinguishing means for each cohort: people living with diabetes, and the nondiabetic controls. Thus, we can display estimated absolute differences between means of the notional combined group (as referent ‘pooled’ mean of zero at each shear stress level) vs model-estimated means for each of the diabetes cohort and nondiabetic controls. For the graphical representation, we back-transformed these shifts and graphed them relative to the effective-as-modeled “pooled mean” of zero (Figure 2A inset). We back-transformed to the original elongation index scale intentionally, to match conventional deformability curves^3,4^.

***Primary Outcome: Approach to Secondary Analyses***

For less-model-dependent graphical and numerical comparisons specific either to: (i) particular shear-stress-levels (including raw-scale shifts in distribution, [Figure 2B](#_Figure_2B)), or (ii) shear-stress-gradient-modeling-derived quantities (including EImax and SS_1/2_, [Figure 2C,](#_Figure_2C) with underlying observations and means depicted using dotplots in Supplementary Figures 1A-B), we have included such secondary analysis findings using distribution-free inferential methods. These include group-specific mean and standard deviation (SD), and relative shifts in distribution between groups (Wilcoxon-Mann-Whitney nonparametric tests with accompanying point and interval estimates for location-shift, Hodges-Lehmann estimates using the [wilcox.test function in R](https://cran.r-project.org/)).

These summaries allowed us to further explore that the primary study readout – the differences in RBCD curves in both groups in [Figure 2A’s](#_Figure_2A) ‘unadjusted’ p-value – was not subject to bias due to group differences in potentially confounding factors (per the highly-significant range of ‘adjusted’ p-values in [Figure 2A](#_Figure_2A)). Our select set of participant characteristics included baseline demographic features or diabetes-status-differing clinical covariates (Table 1) or covariates significantly associated with SS_1/2_ as an univariate measure of RBCD ([Figure 3](#_Figure_3), using the [lm & glm functions in R](https://cran.r-project.org/)). As mean corpuscular volume (MCV) is among these diabetes-status-differing clinical covariates, we further provide graphical and inferential summaries of key remaining blood measures (MCHC% and Hemoglobin), with Ohyper juxtaposed with these underlying observations and respective means, depicted using a scatterplot in Supplementary Figure 2A (with Pearson’s correlation coefficient estimates and p-value) and dotplots (at times stratified by sex) in Supplementary Figures 2B-F (Welch’s *t*-test p-values indicated). Similarly, as diabetes-important measures like plasma glucose and HemoglobinA1c were among such covariates, similar type summaries are given in Supplementary Figures 3A-D, augmented by a table of RBCD derived measure (half-maximal shear stress) tertile subgroups.

***Approach to Exploratory Analyses***

Exploratory analysis assessed how RBCD measures vary jointly with both diabetes status and the following sets of RBC physiologic/rheologic measures, noting each set entails mutual statistical dependence among its component members: (i) osmotic-gradient elongation indices and osmolality measures ([Figures 4](#_Figure_4)); (ii) osmotic fragility (Omin; [Figures 5](#_Figure_5)); and (iii) hemoglobin-oxygen dissociation (p50; [Figures 6](#_Figure_6)).

To determine how the cross-sectional study’s tertiary outcomes compare in study cohorts, we used linear correlations of Box-Cox transformed continuous values (Pearson’s product-moment correlation coefficient, corresponding to linear regression modeling) while also considering distribution-free methods, described above, for osmotic-gradient and osmolality measures (Figures 4[B](#_Figure_4B)-[C](#_Figure_4C), with interpretations referent to the scheme visualized in [Figure 4A](#_Figure_4A)). Additionally, for (ii)-(iii) we explored stratification by RBCD subgroups: rather than strict dichotomization or presuming linearity or stratifying by quantiles that result in 4 or more strata, we employed tertile-based strata with moderately-sized subgroups for some appeal to large-sample theory underlying inferential statistics (n $\approx$ 20 to 35; see, for example, Lenth, R. 2001, “Some Practical Guidelines for Effective Sample-Size Determination,” The American Statistician, 55, 187–193).^11^ Thus, graphically apparent trends in continuous-measure distributions are statistically tested for shifts in means across SS_1/2_ tertile subgroups within the diabetes cohort and nondiabetic controls (Figures 5[C-D](#_Figure_5C), 6[B-C,](#_Figure_6B-C) with underlying estimates listed in Supplementary Tables). Overall bivariate trends with RBCD were graphically shown alongside nested-model reduction inferences (Figures [5E](#_Figure_5E) & [6D](#_Figure_6D)), displaying extent to which RBCD tertiles—representing a *third* variable—explain bivariate variation under the assumed regression model in an accompanying ‘two-directional’ relationship plot, obtained using marginal models for repeated-measures-bivariate-outcome vectors. Each unique pairing of SS_1/2_ and Omin or p50 had its own Box-Cox-transformed values, fitted via generalized estimating equations with working exchangeable correlation models and empirical (sandwich-estimator) based standard errors to yield Wald-type test statistics.^5^

All analyses were conducted assuming: 5% significance; missing values were assumed to be missing at random, using available-case analyses (random subsets of cohort blood samples underwent additional rheology measures, and the relevant amount of demographic covariate balance was assessed using standardized mean differences per methods recently refined^6^; see Supplementary Figures 4); and using R version 4.0 or higher ([www.r-project.org](http://www.r-project.org/)—with additional [packages](https://cran.r-project.org/web/packages/index.html) not mentioned above—for data preparation/visualization—car, chest, DescTools, DHARMa, exact2x2, exactci, ggplot2, glmnet, haven, Hmisc, MASS, mmmgee, patchwork, plotly, quantreg, tidyverse), with occasional ancillary corroboration by SAS v9.4 (as mentioned above, PROC MIXED).

## ***Rationale for Above Approaches, Expanded***

To minimize possibilities of false positive findings, we emphasized repeated measures analyses wherever possible, focusing our analyses on how these ‘multivariate’ panels of physiologic measures—by design—entail some degree of mutually-statistically-dependent co-variation, in a probabilistic sense. As such an approach appears (to the best of our knowledge) to be relatively unprecedented, we prioritized repeated-measures analyses that may be the most robust to mis-specification of such multivariate dependency: marginal models fitted by generalized estimating equations (or GEEs), in which inferences about mean-model features (such as difference between cohorts, even adjusting for other covariates) will still maintain their anticipated inferential operating characteristics even if the ‘working model’ for statistical dependence among repeated measures—or ‘working correlation’ model—is not close to the true (actual) data generating mechanism’s underlying dependence. These Such models have been in use over three decades throughout biomedical and epidemiologic research involving repeated-measures or clustered/longitudinal extensions to conventional approaches (such as the more typically encountered methods that look at only a single outcome at one time). Additional information about how these marginal models fitted via GEEs work in practice is described by others.^7^

Our analyses were conducted with an overarching goal of ensuring that the panoply of findings were robust to both the choice of methods used and how each method’s inherent modeling assumptions can not only be closely met in this current study’s sample, but also be within sampling variability of later follow-on studies’ sample data (thus, all such estimates and accompanying rationales have been included in the report for sake of replicability by other investigative teams). That said, we report our initial single-variable explorations that intentionally made the fewest assumptions possible, i.e., distribution-free or “nonparametric” comparisons. Thus, our foremost counterpart to primary analysis includes one of the methods most widely used in in comparing groups – Wilcoxon (or Mann-Whitney) tests. Notably, these can themselves yield an estimate of the shift in an outcome variable’s distribution in terms of location, the Hodges-Lehmann estimate with accompanying uncertainty quantification via 95% confidence intervals. Given their immediate accessibility within open-source free statistical software, we emphasize these estimates among all secondary analyses since they will be useful for follow-on studies’ own design-stage planning for the purpose of power and sample size. Were the exposure of interest assigned by investigators, more distribution-free methods could have been used. In addition, such methods would simultaneously accommodate our relatively modest (sub-)cohort sizes, as we would be able to leverage study design features such as randomization (such as methods in, for example, Pesarin & Salmaso 2010)^8^. Yet this all presupposes that inferences done one variable at a time may not fall prey to inflated false positive rates in the study’s findings, *overall*, especially as the variables (and each variable’s comparisons) are clearly interdependent statistically*.* We therefore needed to mitigate the chances that inferences from such single-variable-at-a-time analyses may not accommodate intrinsically-dependent variables’ co-variation adequately. Thus, we were required to transform many of these measures for use in repeated-measures modeling. Using the longstanding practice of Box-Cox power transformation analysis, we thus went beyond univariate comparisons into (semi)parametric repeated-measures regression, itself relying on near-normality in its residual variation at each covariate level, to have our findings properly account for the inherent mutual statistical dependence among these red blood cell measures. We employed a decades-longstanding family-of-power-transformations approach from Box & Cox^9^ to yield values that achieve near normality. The approach allows one to simultaneously consider both study-sample-ascertainment features (such as diabetes status) alongside other potentially confounding covariates that may well impact red blood cell measures (such as age in years, whether linearly or in other non-linear forms). We used this approach, such that resulting repeated-measures models are more likely to be tenable in yet-to-be realized studies’ samples. Now, with Box-Cox transformations employed not only in the primary analyses but also in these exploratory ones, this work can again leverage repeated-measures models (to undergird how a bivariate distribution may be well-predicted by tertiles of a single RBCD deformability curve derived measure like half maximal shear stress). Resulting exploratory modeling findings thus have a similar rationale as that adopted for the primary analyses. For more behind the rationale on employing Box-Cox analyses ahead of modeling, please refer to the properties of such transformations as described outlined in chapter 8 “Mathematical Aspects of Transformation” of the longstanding exploratory data analysis text from 1983, *Understanding Robust and Exploratory Data Analysis*, edited by David C. Hoaglin, Frederick Mosteller, & John W. Tukey.^10^

We have intentionally included more than one approach to comparing measures even within exploratory analyses for important reasons that attend these relatively novel findings. Not only do we employ two-variable-at-a-time correlation inference alongside bivariate scatterplots, but we also employ repeated-measures modeling inference about a single (third) RBCD measure as annotated in the ‘two-directional’ relationship plots. This is all to assure that findings would be less susceptible to false positive conclusions predicated on one particular (perhaps inadvertently chosen) analysis approach, especially as (a) these measures’ distributions are not inherently Gaussian on their raw scale, and (b) some amount of transformation is required to take advantage of the necessarily more sophisticated modeling that leverages all the statistically-dependent information in the full panel of each subset (i)-(iii) of RBC rheology measures, as outlined under Approach to Exploratory Analyses above.

REFERENCES

1. Zeger, S.L. & Liang, K.Y. Longitudinal data analysis for discrete and continuous outcomes. *Biometrics* **42**, 121-130 (1986).

2. Ziegler, A., Blettner, M., Kastner, C. & Chang-Claude, J. Identifying influential families using regression diagnostics for generalized estimating equations. *Genet Epidemiol* **15**, 341-353 (1998).

3. Baskurt, O.K.*, et al.* Comparison of three commercially available ektacytometers with different shearing geometries. *Biorheology* **46**, 251-264 (2009).

4. Parrow, N.L.*, et al.* Measuring Deformability and Red Cell Heterogeneity in Blood by Ektacytometry. *Jove-J Vis Exp* (2018).

5. Zhao, L.P., Prentice, R.L. & Self, S.G. Multivariate Mean Parameter Estimation by Using a Partly Exponential Model. *Journal of the Royal Statistical Society Series B: Statistical Methodology* **54**, 805-811 (1992).

6. Greifer, N. Cobalt: Covariate Balance Tables and Plots. R package version 4.5.5.9000. (2024).

7. Hanley, J.A., Negassa, A., Edwardes, M.D. & Forrester, J.E. Statistical analysis of correlated data using generalized estimating equations: an orientation. *Am J Epidemiol* **157**, 364-375 (2003).

8. *Permutation Tests for Complex Data*, (2010).

9. Box, G.E.P. & Cox, D.R. An Analysis of Transformations. *Journal of the Royal Statistical Society Series B: Statistical Methodology* **26**, 211-243 (1964).

10. Hoaglin, D.C. John W. Tukey and Data Analysis. *Statistical Science* **18**, 311-318 (2003).

11. Lenth, R.V. Some Practical Guidelines for Effective Sample Size Determination. *The American Statistician* **55**, 187-193 (2001).
