# Supplementary material for: Altered RBC deformability in diabetes: clinical characteristics and RBC pathophysiology
Source: Cardiovasc Diabetol. 2024 Oct 18;23:370. doi: 10.1186/s12933-024-02453-2 (PMC11490132; doi:10.1186/s12933-024-02453-2)
Supplement: Supplementary file 2 — Supplementary Material 2 [file 12933_2024_2453_MOESM2_ESM.pptx]

## Slide 1
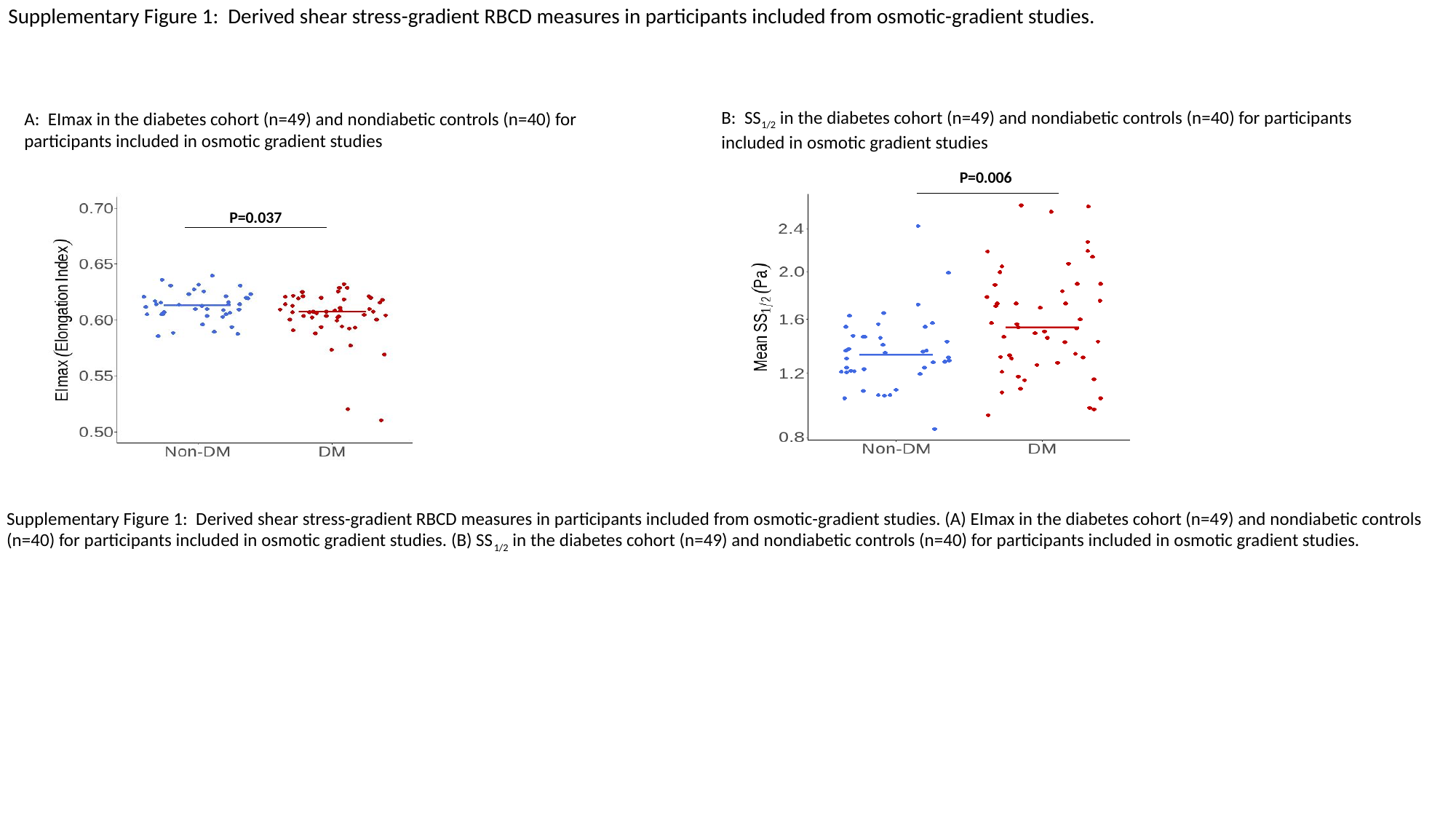

Supplementary Figure 1: Derived shear stress-gradient RBCD measures in participants included from osmotic-gradient studies.
B: SS1/2 in the diabetes cohort (n=49) and nondiabetic controls (n=40) for participants included in osmotic gradient studies
A: EImax in the diabetes cohort (n=49) and nondiabetic controls (n=40) for participants included in osmotic gradient studies
P=0.006
P=0.037
Supplementary Figure 1: Derived shear stress-gradient RBCD measures in participants included from osmotic-gradient studies. (A) EImax in the diabetes cohort (n=49) and nondiabetic controls (n=40) for participants included in osmotic gradient studies. (B) SS1/2 in the diabetes cohort (n=49) and nondiabetic controls (n=40) for participants included in osmotic gradient studies.

## Slide 2
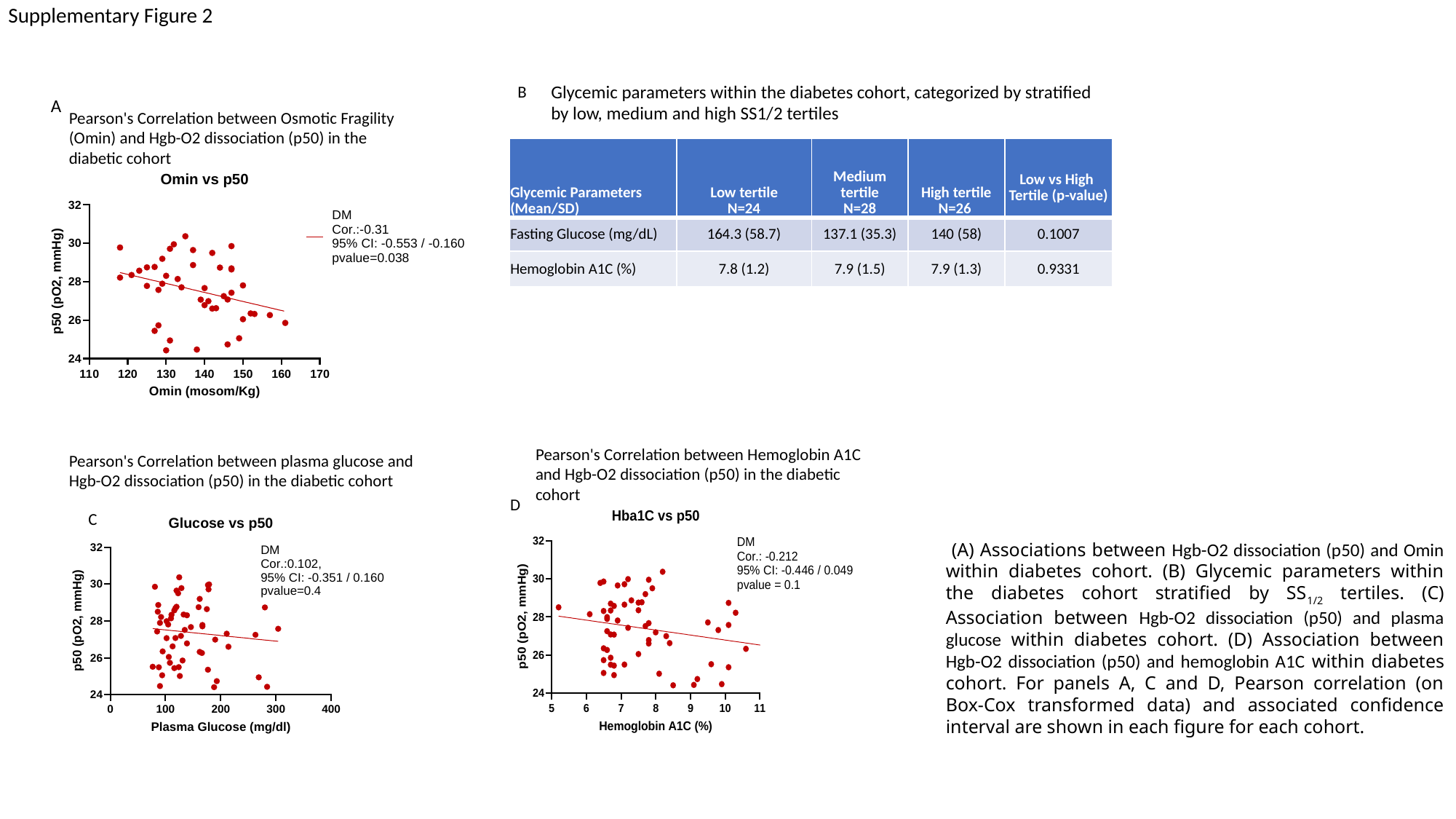

Supplementary Figure 2
Glycemic parameters within the diabetes cohort, categorized by stratified by low, medium and high SS1/2 tertiles
B
A
Pearson's Correlation between Osmotic Fragility (Omin) and Hgb-O2 dissociation (p50) in the diabetic cohort
| Glycemic Parameters (Mean/SD) | Low tertile N=24 | Medium tertile N=28 | High tertile N=26 | Low vs High Tertile (p-value) |
| --- | --- | --- | --- | --- |
| Fasting Glucose (mg/dL) | 164.3 (58.7) | 137.1 (35.3) | 140 (58) | 0.1007 |
| Hemoglobin A1C (%) | 7.8 (1.2) | 7.9 (1.5) | 7.9 (1.3) | 0.9331 |
Pearson's Correlation between Hemoglobin A1C and Hgb-O2 dissociation (p50) in the diabetic cohort
Pearson's Correlation between plasma glucose and Hgb-O2 dissociation (p50) in the diabetic cohort
D
C
 (A) Associations between Hgb-O2 dissociation (p50) and Omin within diabetes cohort. (B) Glycemic parameters within the diabetes cohort stratified by SS1/2 tertiles. (C) Association between Hgb-O2 dissociation (p50) and plasma glucose within diabetes cohort. (D) Association between Hgb-O2 dissociation (p50) and hemoglobin A1C within diabetes cohort. For panels A, C and D, Pearson correlation (on Box-Cox transformed data) and associated confidence interval are shown in each figure for each cohort.

## Slide 3
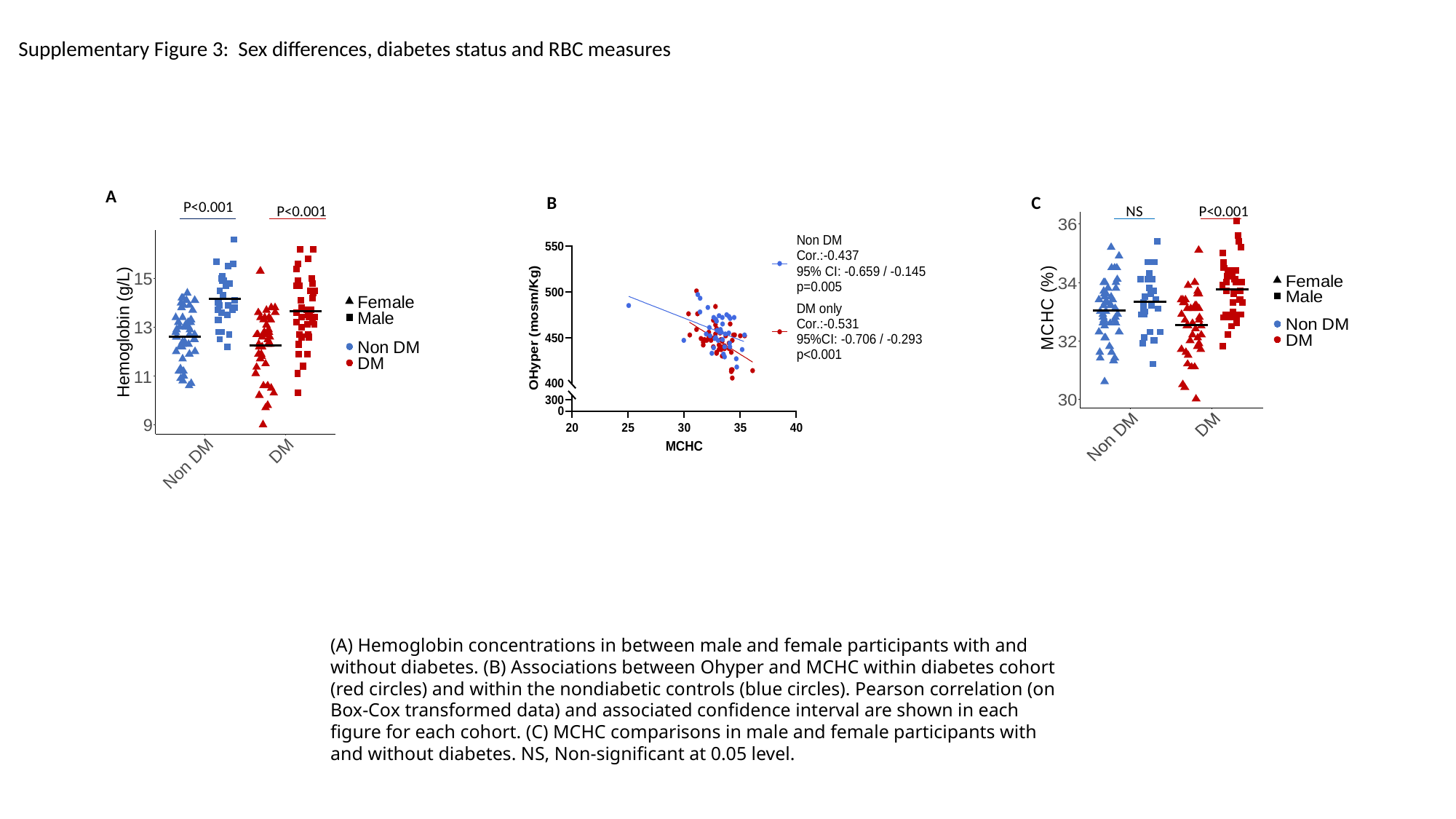

Supplementary Figure 3: Sex differences, diabetes status and RBC measures
A
B
C
P<0.001
NS
P<0.001
P<0.001
(A) Hemoglobin concentrations in between male and female participants with and without diabetes. (B) Associations between Ohyper and MCHC within diabetes cohort (red circles) and within the nondiabetic controls (blue circles). Pearson correlation (on Box-Cox transformed data) and associated confidence interval are shown in each figure for each cohort. (C) MCHC comparisons in male and female participants with and without diabetes. NS, Non-significant at 0.05 level.

## Slide 4
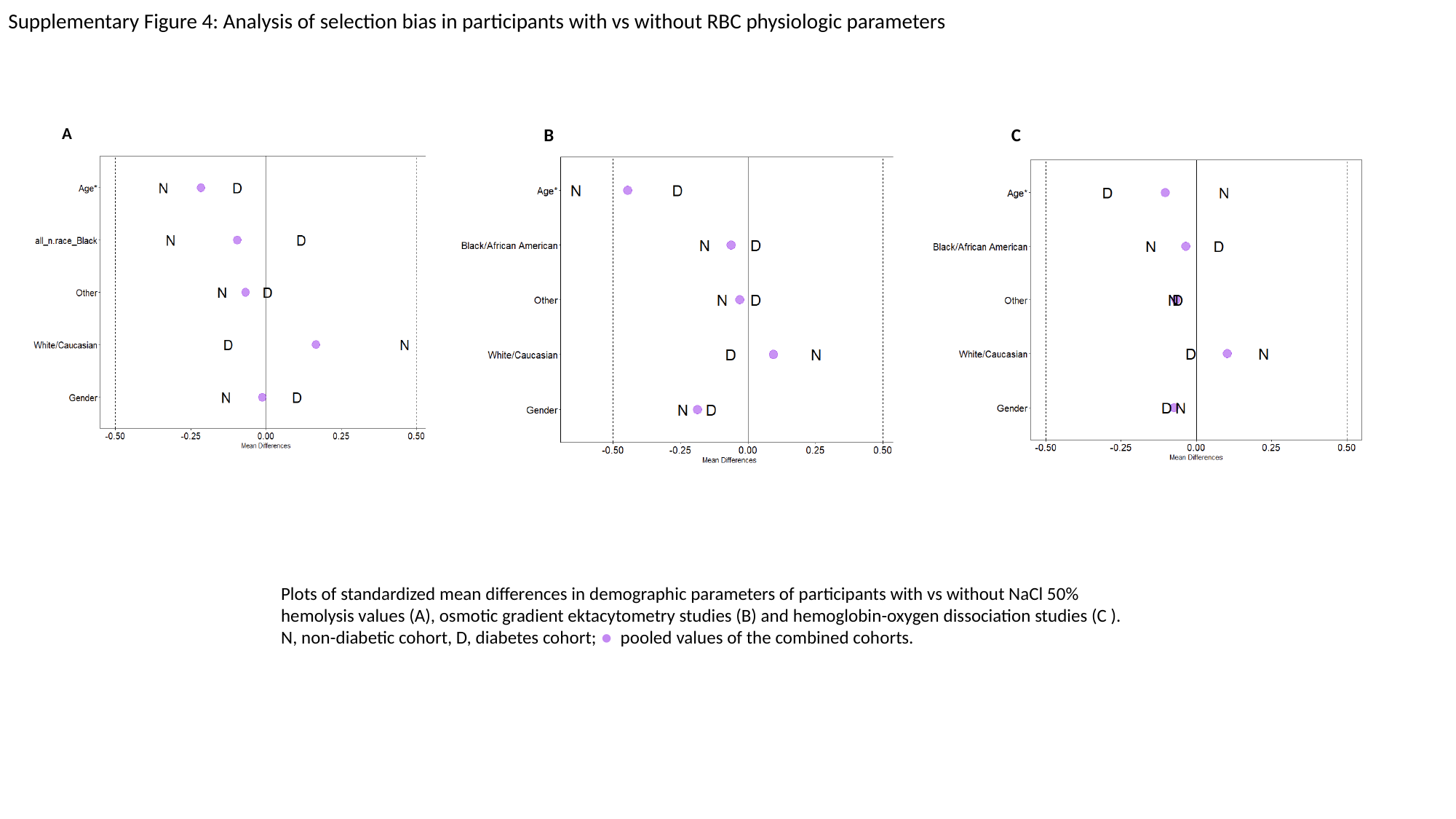

Supplementary Figure 4: Analysis of selection bias in participants with vs without RBC physiologic parameters
A
B
C
Plots of standardized mean differences in demographic parameters of participants with vs without NaCl 50% hemolysis values (A), osmotic gradient ektacytometry studies (B) and hemoglobin-oxygen dissociation studies (C ). N, non-diabetic cohort, D, diabetes cohort; ● pooled values of the combined cohorts.

## Slide 5
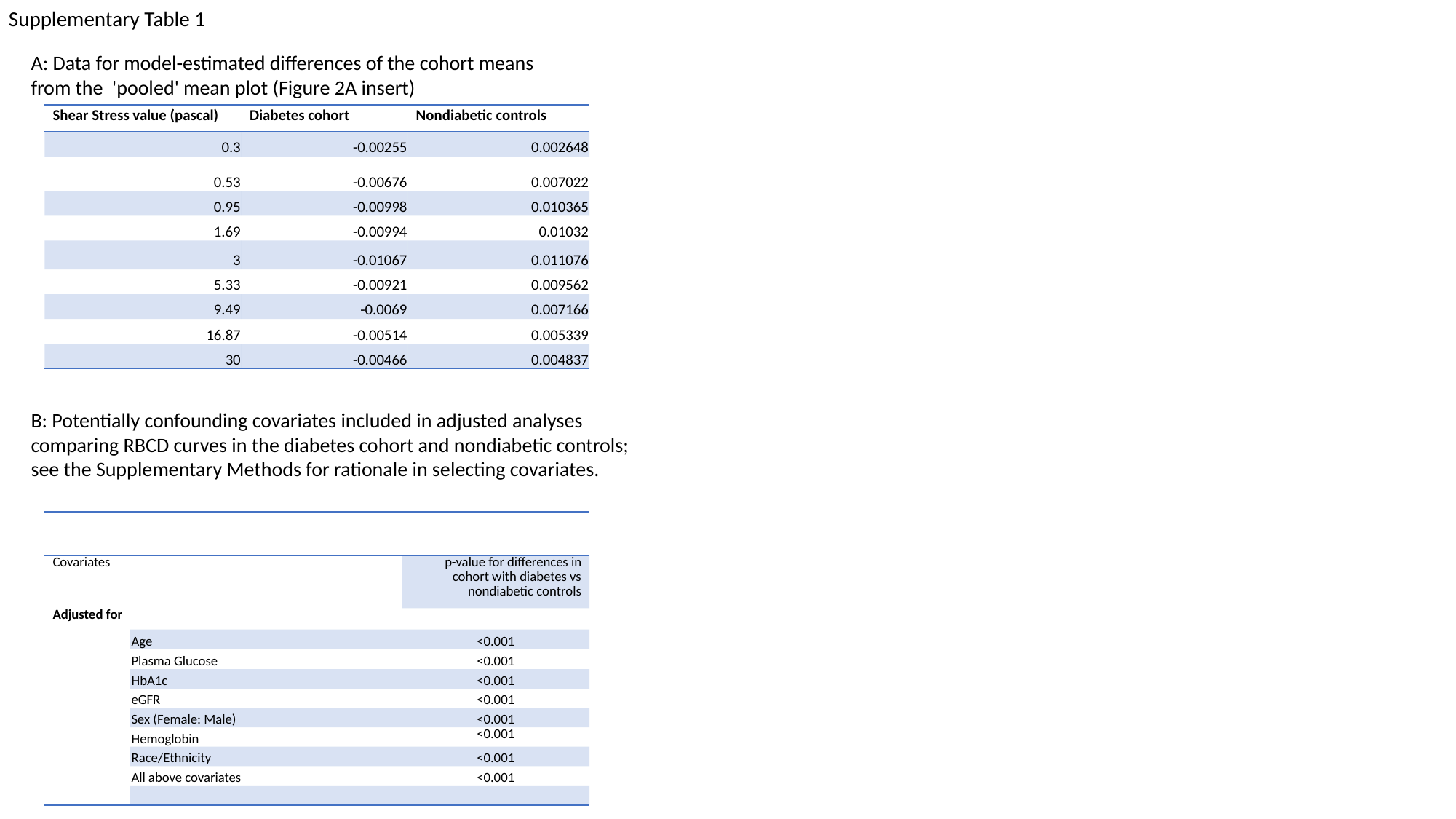

Supplementary Table 1
A: Data for model-estimated differences of the cohort means from the 'pooled' mean plot (Figure 2A insert)
| Shear Stress value (pascal) | Diabetes cohort | Nondiabetic controls |
| --- | --- | --- |
| 0.3 | -0.00255 | 0.002648 |
| 0.53 | -0.00676 | 0.007022 |
| 0.95 | -0.00998 | 0.010365 |
| 1.69 | -0.00994 | 0.01032 |
| 3 | -0.01067 | 0.011076 |
| 5.33 | -0.00921 | 0.009562 |
| 9.49 | -0.0069 | 0.007166 |
| 16.87 | -0.00514 | 0.005339 |
| 30 | -0.00466 | 0.004837 |
B: Potentially confounding covariates included in adjusted analyses comparing RBCD curves in the diabetes cohort and nondiabetic controls; see the Supplementary Methods for rationale in selecting covariates.
| | | |
| --- | --- | --- |
| Covariates | | p-value for differences in cohort with diabetes vs nondiabetic controls |
| Adjusted for | | |
| | Age | <0.001 |
| | Plasma Glucose | <0.001 |
| | HbA1c | <0.001 |
| | eGFR | <0.001 |
| | Sex (Female: Male) | <0.001 |
| | Hemoglobin | <0.001 |
| | Race/Ethnicity | <0.001 |
| | All above covariates | <0.001 |
| | | |

## Slide 6
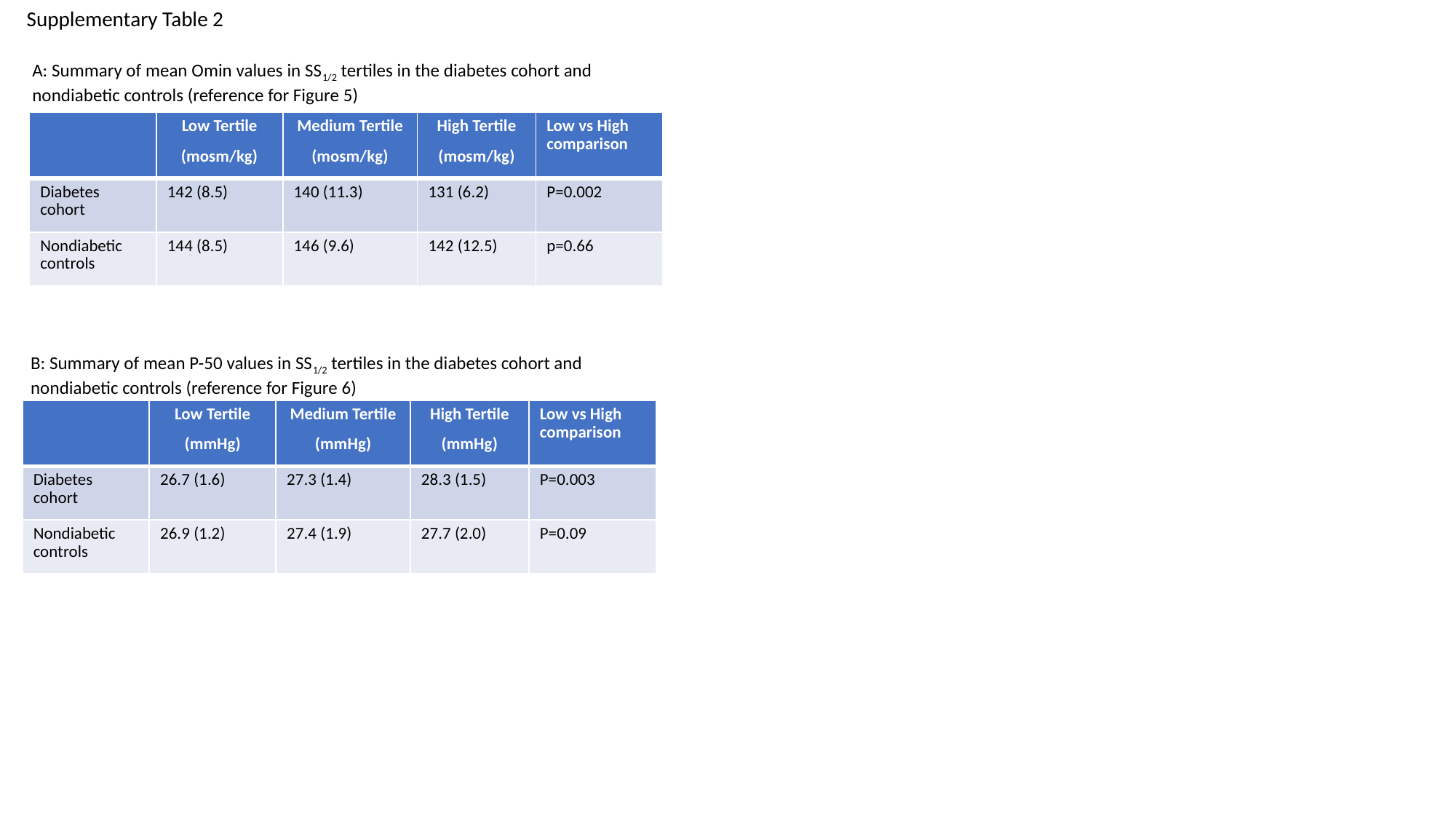

Supplementary Table 2
A: Summary of mean Omin values in SS1/2 tertiles in the diabetes cohort and nondiabetic controls (reference for Figure 5)
| | Low Tertile (mosm/kg) | Medium Tertile (mosm/kg) | High Tertile (mosm/kg) | Low vs High comparison |
| --- | --- | --- | --- | --- |
| Diabetes cohort | 142 (8.5) | 140 (11.3) | 131 (6.2) | P=0.002 |
| Nondiabetic controls | 144 (8.5) | 146 (9.6) | 142 (12.5) | p=0.66 |
B: Summary of mean P-50 values in SS1/2 tertiles in the diabetes cohort and nondiabetic controls (reference for Figure 6)
| | Low Tertile (mmHg) | Medium Tertile (mmHg) | High Tertile (mmHg) | Low vs High comparison |
| --- | --- | --- | --- | --- |
| Diabetes cohort | 26.7 (1.6) | 27.3 (1.4) | 28.3 (1.5) | P=0.003 |
| Nondiabetic controls | 26.9 (1.2) | 27.4 (1.9) | 27.7 (2.0) | P=0.09 |
